# Exosomes from Hepatitis C Infected Patients Transmit HCV Infection and Contain Replication Competent Viral RNA in Complex with Ago2-miR122-HSP90

**DOI:** 10.1371/journal.ppat.1004424

**Published:** 2014-10-02

**Authors:** Terence N. Bukong, Fatemeh Momen-Heravi, Karen Kodys, Shashi Bala, Gyongyi Szabo

**Affiliations:** Department of Medicine, University of Massachusetts Medical School, Worcester, Massachusetts, United States of America; University of Alabama at Birmingham, United States of America

## Abstract

Antibodies targeting receptor-mediated entry of HCV into hepatocytes confer limited therapeutic benefits. Evidence suggests that exosomes can transfer genetic materials between cells; however, their role in HCV infection remains obscure. Here, we show that exosomes isolated from sera of chronic HCV infected patients or supernatants of J6/JFH1-HCV-infected Huh7.5 cells contained HCV RNA. These exosomes could mediate viral receptor-independent transmission of HCV to hepatocytes. Negative sense HCV RNA, indicative of replication competent viral RNA, was present in exosomes of all HCV infected treatment non-responders and some treatment-naïve individuals. Remarkably, HCV RNA was associated with Ago2, HSP90 and miR-122 in exosomes isolated from HCV-infected individuals or HCV-infected Huh7.5 cell supernatants. Exosome-loading with a miR-122 inhibitor, or inhibition of HSP90, vacuolar H^+^-ATPases, and proton pumps, significantly suppressed exosome-mediated HCV transmission to naïve cells. Our findings provide mechanistic evidence for HCV transmission by blood-derived exosomes and highlight potential therapeutic strategies.

## Author Summary

Since its first isolation and identification in 1989, Hepatitis C virus (HCV), has caused significant disease burden to humans worldwide. So far, there is no vaccine against HCV, and neutralizing antibody therapies to block receptor–mediated transmission of HCV to liver cells have so far achieved limited therapeutic benefits. This indicates that HCV can transmit infection via receptor-independent mechanisms. Evidence suggests that small host extracellular vesicles (exosomes) can mediate receptor-independent transfer of genetic material between cells, though their role in HCV transmission remains uncertain. Here, we found that the HCV virus can utilize host exosomes to transmit infection to naïve liver cells, even in the presence of potent blocking anti-HCV receptor antibody treatments. Additionally, we identified alternative treatment strategies that can block host exosomes from transmitting HCV infection. Our study provides novel insights to an alternative mechanism of HCV transmission that can compromise anti-HCV immune therapies and proposes potential therapeutic approaches to block exosome-mediated transmission of HCV infection.

## Introduction

Hepatitis C virus (HCV) infection is one of the leading causes of liver disease with over 170 million individuals chronically infected worldwide [1], [2]. Severe complications including fibrosis, cirrhosis, and hepatocellular carcinoma are among the long-term effects of HCV infection, making liver transplantation the ultimate choice of treatment for advanced liver disease [3]. Even with successful liver transplantation, patients face eminent HCV re-infection of the newly transplanted liver. Recent therapies with anti-HCV E1-E2 or other neutralizing antibodies that attempted to block HCV transmission achieved only limited success [4]–[7].

HCV is a positive-sense single-stranded RNA enveloped virus of the Flaviviridae family. The 9.6 kb HCV genomic RNA encodes a single polypeptide that is proteolytically cleaved to structural (core, E1, and E2) and non-structural (p7, NS2, NS3, NS4A, NS4B, NS5A and NS5B) HCV viral proteins [8]. The HCV viral envelope E1 and E2 proteins engage numerous host cell proteins for viral entry including CD81 [9]–[11]. CD81 interaction with HCV E1/E2 is critical in HCV entry and anti-CD81 or anti-E1/E2 antibodies have been shown to block HCV virus entry [7], [12]. Given the importance of these viral envelope proteins in regulating HCV infection, numerous immune therapies have been developed to specifically target and/or neutralize HCV envelope proteins [7], [13]–[15]. Targeted antibody therapies have offered limited success in preventing liver allograft infection by HCV. Recently, a potent human-derived monoclonal antibody was demonstrated to effectively prevent and treat HCV1 infection in chimpanzees [7]. However, the same antibody was not completely effective in humans [7], raising the possibility of other mechanisms of virus entry into hepatocytes. Previous reports have suggested receptor independent transmission of HCV [6], [16], though the precise mechanisms or possible therapeutic strategies remain to be explored.

Exosomes are a subpopulation of extracellular vesicles that originate from multivesicular bodies (MVBs), ranging from 40–150 nm in size and are produced by most cell types. These vesicles can be detected in blood, urine, and other body fluids [17]. Exosomes can modulate signal transduction, antigen presentation to T-cells, and transmission of genetic material between cells [18]. Over the past decade, a great body of evidence shows that exosomes can be secreted into the extracellular space and can mediate indirect cell-to-cell communication by transferring bio macromolecules, functional proteins, and RNAs between cells [19], [20].

HCV infection occurs via cell free virus and direct cell-to-cell transmission [6]. Indirect cell-to-cell transmission is another pathway to consider. Previously, HCV viral RNA has been identified in supernatant of HCV-SGR cells [21] and exosome-like structures have been detected in the supernatant of HCV infected cells [22] and in the plasma of HCV-infected patients [23]. Recently, Dreux et al (2013) showed that HCV-RNA-containing exosomes can mediate transfer of RNA to co-cultured plasmacytoid dendritic cells (pDCs) and trigger the production of type I interferon (IFN) in vitro [24].

Here we tested the hypothesis that exosomes derived from HCV infected hepatocytes or from the sera of HCV infected patients carry viral RNA and exploit the cellular exosomal delivery system to mediate receptor-independent HCV transmission to hepatocytes. We found that exosomes derived from HCV infected Huh7.5 cells and HCV infected patients contained HCV RNA that induced active infection in primary human hepatocytes (PHH). These exosomes were rich in replication-competent HCV-RNA in complex with miR-122, Ago2, and HSP90; and mediated HCV transmission independent of CD81, SB-RI and APOE. Mechanistically, functional inhibition of miR-122, HSP90 or modification of cellular micro-environmental pH using a vacuolar-type H+-ATPase (V-ATPase) and proton pump inhibitor significantly suppressed the capacity of exosomes to mediate HCV transmission.

## Results

### Separation of HCV infected exosomes from free HCV viruses and characterization of exosomes

**Figure 1:**
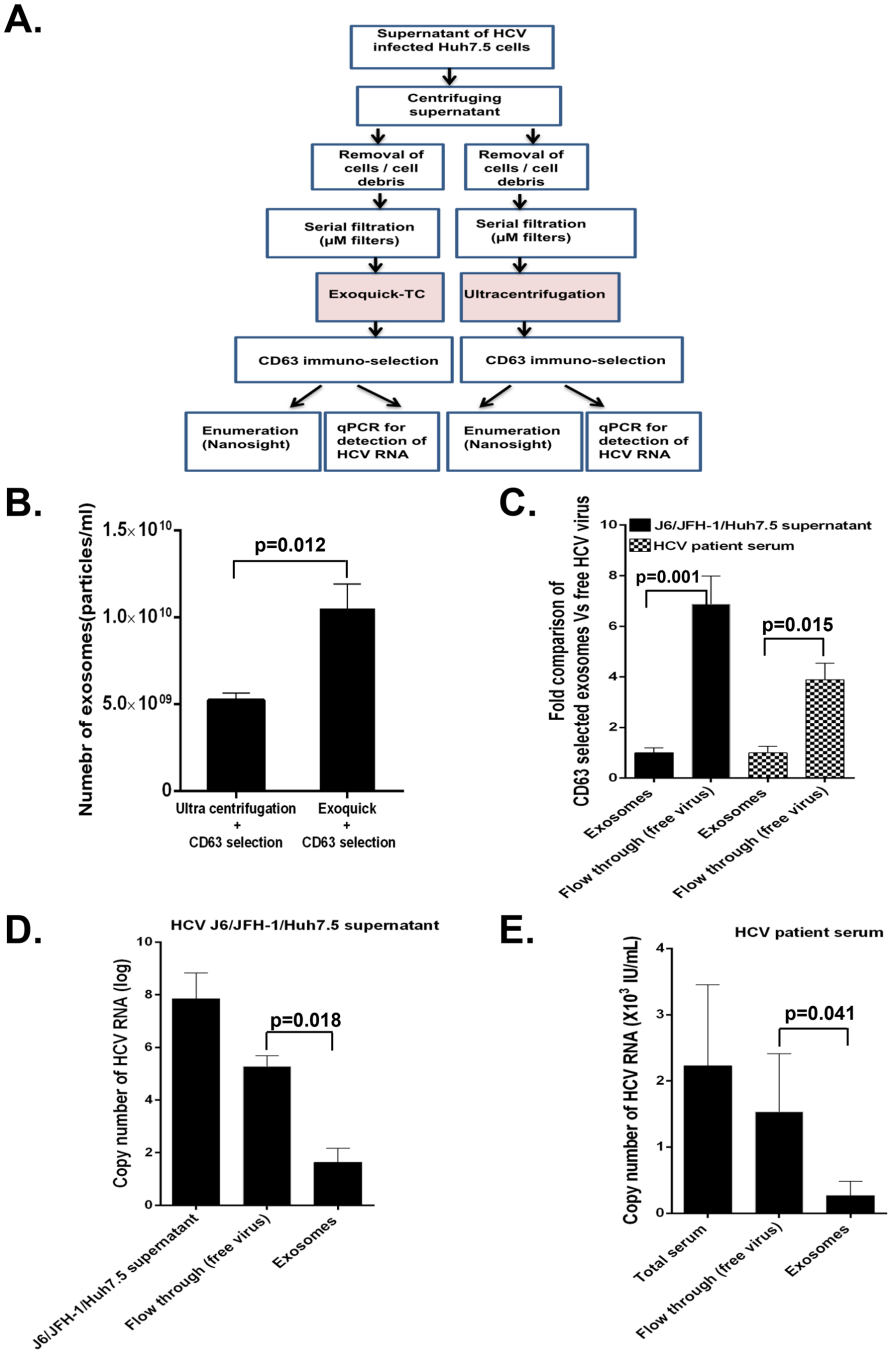
Assessing the efficiency of exosome isolation protocol. (A) Schematic experimental flow chart to compare the efficiency of two exosome isolation methods from cell culture supernatants, Exoquick+CD63 immuno-magnetic selection and ultracentrifugation+CD63 immuno-magnetic selection for recovery of exosomes. (B) Exosomes were isolated by either Exoquick+CD63 immuno-magnetic selection or ultracentrifugation+CD63 immuno-magnetic. Isolated exosomes were quantified using Nanosight (NTA). (C) Total CD63 selected exosomes and cell free HCV virus was isolated from cell culture supernatants of HCV J6/JFH-1 infected Huh7.5 cells and HCV infected serum as detailed in the methods. Total number of exosomes and HCV viral particles isolated were quantified using NanoSight. Data is expressed as fold comparison for three independent repeat cell culture experiments and 5 serum samples from HCV infected patients. (D) Total RNA was extracted from total culture supernatants of HCV J6/JFH-1 infected Huh7.5 cells, flow through samples (free HCV virus) and CD63 selected exosomes (HCV exosomes) and analysed for HCV RNA content by quantitative real-time PCR. (E) Total RNA was extracted from HCV infected patient serum, flow through (free HCV patient virus) and CD63 selected HCV patient exosomes and analysed for HCV RNA content by quantitative real-time PCR. Results are representative of 4 repeat experiments and 6 HCV infected patient samples expressed as mean + SEM with p<0.05 considered statistically significant using ANOVA analysis and Mann-Whitney U test using GraphPad prism 5.0 software.

Given our interest to investigate the capacity of exosomes derived from HCV J6/JFH-1-infected Huh7.5 cells and serum of HCV infected patients to mediate active transmission, we had to efficiently separate exosomes from the free HCV virus. Because HCV virions and exosomes have very similar sizes and densities, the traditional ultracentrifugation and sucrose gradient isolation method is insufficient for isolating pure exosomes free of virus contamination. To overcome this limitation, we optimized a CD63 immuno-magnetic isolation method to purify exosomes from cell culture supernatants of HCV J6/JFH-1 infected Huh7.5 cells and sera of HCV infected patients. Briefly, after serial filtration (1 µm, 0.44 µm and 0.22 µm) of supernatants, exosomes were initially isolated using Exoquick. To further purify exosomes and exclude other microparticles or free HCV contamination, Exoquick-isolated exosomes were subjected to immuno-magnetic selection with CD63, a selection marker of exosomes. This protocol was verified by analysis for other exosomal markers by western blotting (CD9 and CD81), electron microscopy, and Nanoparticle tracking analysis (NTA) (Figures S1A, S1B & S1C). The Exoquick-CD63 immuno-magnetic selection procedure recovered more exosomes compared to, ultracentrifugation-CD63 immuno-selection of exosomes (Fig. 1A & 1B); based on this observation we used the Exoquick followed by CD63 immuno-magnetic selection for subsequent experiments. We observed that in a fixed total volume there were significantly more free HCV viral particles compared to the number of exosome particles in HCV J6/JFH-1 infected Huh7.5 cell supernatants [approximately 7∶1 ratio] and in HCV infected patient serum samples [approximately 4∶1 ratio] (Fig. 1C). We also found higher HCV viral copy numbers in the free virus fraction compared to exosomes in J6/JFH-1 infected Huh7.5 cell supernatants (Fig. 1D) and HCV patients' serum (Fig. 1E). Purified exosomes were analyzed by transmission electron microscopy, demonstrating their vesicular shape and size range between 50 and 100 nm (Figure S1A). Further analysis with NanoSight demonstrated comparable histogram size plots of exosomes from culture supernantants of HCV J6/JFH-1 infected Huh7.5 cells and exosomes from serum of HCV infected patients (Figure S1B & S1C).

**Figure 2:**
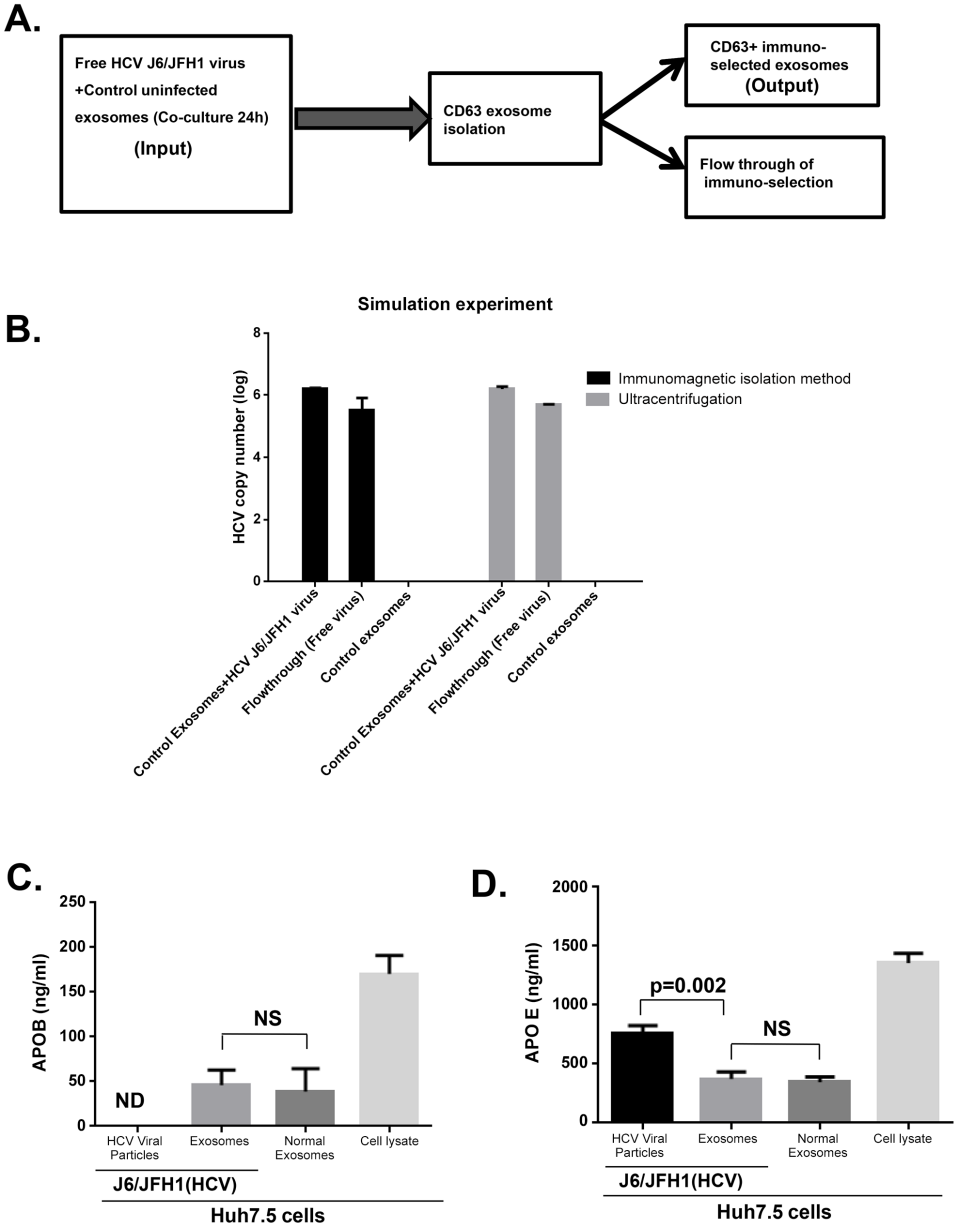
Simulation study ruling out viral carryover of cell free HCV virus and purity of isolated exosome. (A) Comparative **s**chematic flow diagram of exosome isolation by Exoquick and ultracentrifugation with additional CD63 immuno-magnetic selection. (B) Control uninfected exosomes were mixed with free HCV virus suspension and after 24 hour co-culture, samples were divided into two portions for (1) total RNA extraction or (2) immuno-magnetic CD63 isolation of the exosomes followed by total RNA extraction. Extracted RNA was analysed for HCV RNA content by quantitative real-time PCR. Results are representative of 4 indipendent experiments. (C &D) Exosomes and free HCV virus were isolated as detailed in our methods. Equal numbers of isolated exosomes and free HCV virus were then lysed in RIPA protein extraction buffer. Extracted total protein from exosomes and cell free virus as indicated was subjected to APOE and APOB ELISA analysis according to the manufacturers' protocol. Results are representative of 3 independent experiments with p<0.05 considered statistically significant usisng ANOVA analysis with GraphPad prism 5.0 software.

To rule out exosome contamination with free HCV virus, we carried out a simulation experiment mixing cell free HCV virus with uninfected exosomes from Huh7.5 cell culture supernatants for 24 h and re-isolated exosomes with Exoquick followed by CD63 immuno-selection or ultracentrifugation followed by CD63 immuno-selection. The uninfected exosomes exposed to free HCV virus showed no detectable HCV viral RNA while HCV RNA was present in the flow through following immuno-magnetic CD63 selection of exosomes (Fig. 2A & 2B). Further characterization of exosomes and free virus showed that isolated exosomes contained Apolipoprotein B (APOB) which was not present in cell free HCV viruses (Fig. 2C). Apolipoprotein E (APOE) was found to be associated to a large extent with HCV virus fraction and significantly lower in exosomes compared to cell free virus fractions (Fig. 2D). These observations suggest that our purified exosomes were to a large extent devoid of lipo-viral contamination. RNase H treatment to destroy free RNA in the cell free virus concentrate and isolated exosomes from HCV infected Huh 7.5 cells failed to prevent transfer of HCV infection to naïve cells, thus ruling out the possibility of envelope free viral RNA mediating HCV infection. These data indicate that both HCV derived exosomes and HCV virus are resistant to RNase treatment similar to the previous report [25] and still cause productive infection even after RNase treatment (Figure S2).

### Exosomes from serum of HCV infected patients or HCV J6/JFH-1 infected Huh7.5 cells contain HCV RNA, miR-122, Ago2, and HSP90

**Figure 3:**
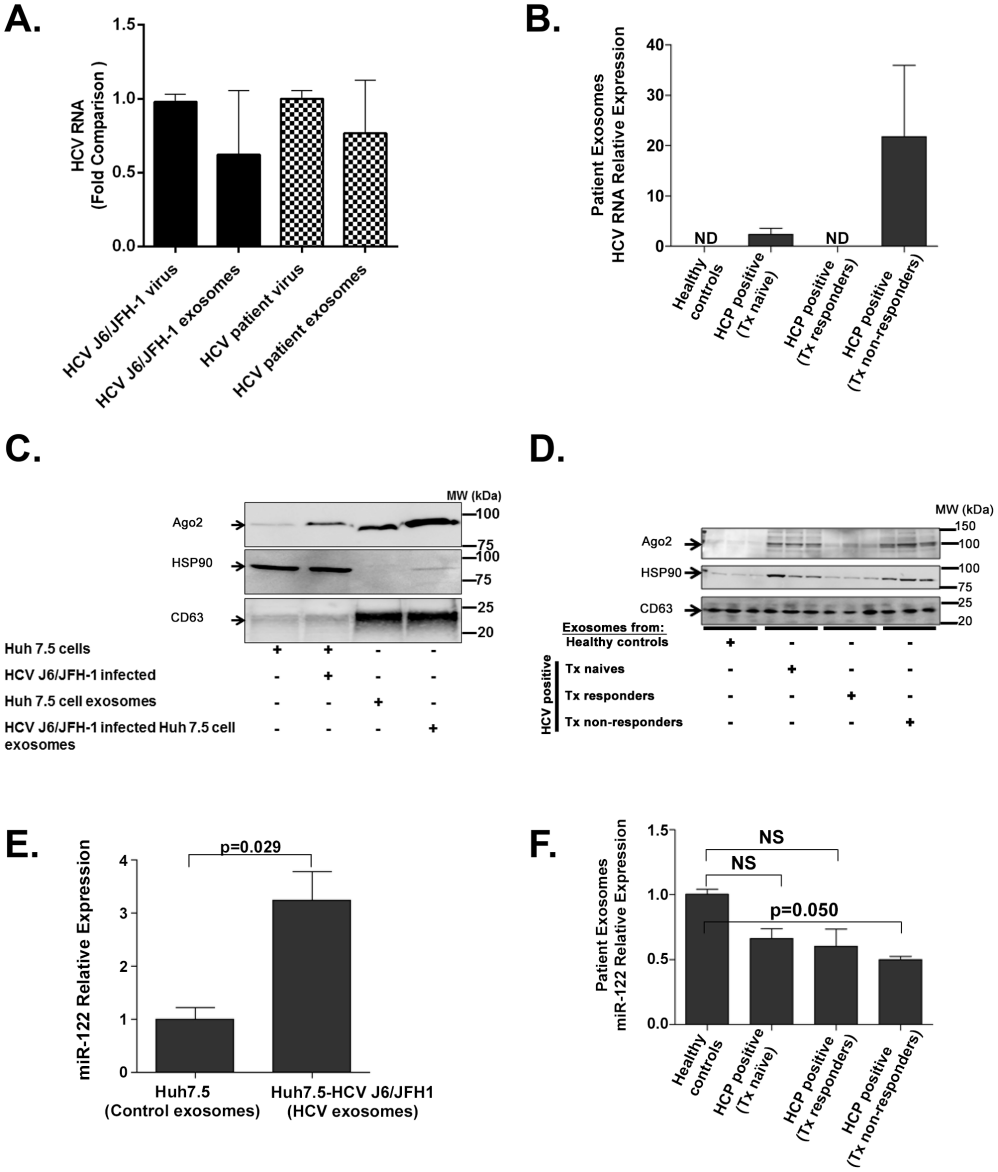
Exosomes from HCV J6/JFH-1 infected Huh7.5 cells and sera of HCV infected patients contain HCV RNA, miR-122, Ago2, and HSP90. Total RNA was extracted from the same number of infectious virus and exosome particles using QIAzole (Qiagen) for lysis then spiked with equal amounts of synthetic C. elegans (cel)-miR-39 and after this step total RNA was extracted using Zymo research Direct-zol RNA MiniPrep kit. 100 ng of total (cell or exosome) RNA was reverse transcribed to cDNA and analysed for HCV RNA using SYBR Green quantitative real-time PCR analysis. Comparative delta-delta ct method was used for determining relative HCV RNA using Cel-miR-39 RNA as normalizing control. (A) HCV RNA expression was analysed in total RNA extracted from HCV J6/JFH-1 virus, HCV exosomes from J6/JFH-1 infected Huh7.5 cell supernatants, free HCV patient virus and HCV patient exosomes. Equal amounts of exogenouse synthetic cel-miR-39 was spiked into each sample during RNA extraction which was later used as normalization control for HCV RNA fold comparison using comparative delta-delta ct method. (B) HCV RNA relative content in the exosomes were measured in different study groups using equal number of serum exosomes from healthy controls, HCV infected treatment naive, HCV infected treatment reponders, and HCV infected treatment non-responder patients determined by quantitative real-time PCR. Equal amounts of exogenouse synthetic cel-miR-39 was spiked into each sample during RNA extraction which was later used as normalization control for HCV RNA fold comparison using comparative delta-delta ct method. (C) 50 µg of protein was used for western-blot analysis of whole cell lysates of Huh7.5 cells, HCV J6/JFH-1 infected Huh7.5 cells, exosomes from Huh7.5 cell supernatants, and exosomes from HCV J6/JFH-1 infected Huh7.5 cell supernatants. (D) Western-blot analysis of exosomal proteins from HCV patients' serum. (E) miR-122 expression of exosomes from Huh7.5 cell supernatants and exosomes from HCV J6/JFH-1 infected Huh7.5 cell supernatants. Equal amounts of exogenouse synthetic cel-miR-39 was spiked into each sample during RNA extraction from exosomes and was later used as normalization control for HCV RNA fold comparison using comparative delta-delta ct method (F) miR-122 profile of exosomes from HCV patient serum samples were analysed by real-time quantitative PCR and using exogenous cel-miR-39 RNA for normalization. Data are expressed as mean + SEM, p<0.05 was considered statistcally significant by Mann-Whitney U test.

First, we established efficient methods for exosome purification using Exoquick followed by CD63-based isolation as described above. These exosomes were devoid of free HCV virus contamination as detailed. Exosomes isolated from sera of some HCV-infected patients or supernatants of HCV J6/JFH1 infected Huh7.5 cells contained comparable HCV RNA content for the same number of free HCV viral particles compared to the same number of HCV exosome particles (Fig. 3A). These observations allowed us to use the same number of infectious HCV viral particles and HCV exosomes for subsequent experiments. Treatment-naïve and non-responder (interferon plus ribavirin) patients with active HCV infection had detectable HCV RNA in serum-derived exosomes (Fig. 3B). In contrast, treatment responders, who cleared HCV infection, showed no detectable HCV in exosomes (Fig. 3B). Additionally, interferon alpha treatment of Huh7.5 cells had no effect on the number of exosomes released from hepatocytes (Figure S3) compared to untreated cells.

Recent studies have consistently demonstrated that miR-122, Ago2, and HSP90 enhance HCV replication [26]–[30]. We found that HCV J6/JFH-1 infected Huh7.5 cells produced exosomes that are enriched in Ago2 and contain barely detectable HSP90 protein compared to control exosomes from Huh7.5 cells (Fig. 3C). Interestingly, exosomes from HCV infected treatment-naïve and treatment non-responder individuals, but not treatment responders, were rich in Ago2 and HSP90 (Fig. 3D) compared to control healthy uninfected individuals. GW182 a RISC complex protein which we recently identified as an enhancer of HCV replication associated with alcohol use [31], was not detected in exosomes in our experimental conditions.

Micro RNA-122, a host factor utilized by HCV for replication, was present in exosomes isolated from both HCV J6/JFH-1 infected Huh7.5 cells and HCV-infected individuals (Fig. 3E & 3F). We observed that exosomes from HCV J6/JFH-1 infected Huh 7.5 cells showed higher levels of miR-122 compared to exosomes from non-infected cells (Fig. 3E), while exosomes from HCV-infected patients contained lower miR-122 levels compared to those from healthy controls (Fig. 3F).

### Exosomes from HCV treatment naïve patients or culture supernatants of HCV J6/JFH-1 infected Huh7.5 cells transmit HCV infection

Exosomes were recently shown to mediate retroviral infection independent of envelope protein-receptor interaction [32]. More recently, exosomes from Huh7.5 infected cells were found to induce type I interferon production in dendritic cells [24].

**Figure 4:**
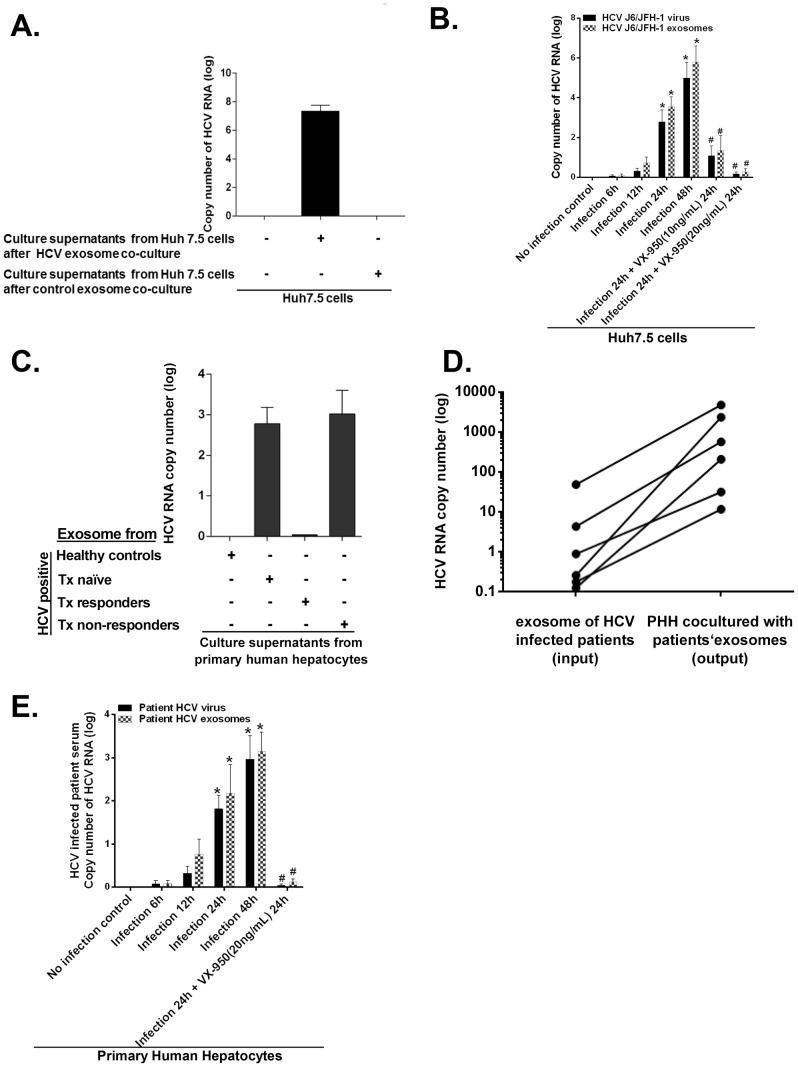
Exosomes from HCV J6/JFH-1 infected Huh7.5 cells transmit HCV infection to the human hepatoma cell line (Huh7.5 cells) and exosomes from HCV infected patients' serum transmit HCV infection to primary human hepatocytes. (A) Exosomes from HCV J6/JFH-1 infected Huh7.5 cell culture supernatants were co-cultured with Huh7.5 cells for 12 h alongside appropriate controls. Exosomes were then washed off and replaced with fresh medium and cells were cultured for another 24 h. Culture supernatant from these exosome-infected Huh7.5 cells were then transferred to naïve Huh7.5 cells and cultured for 24 h. Total RNA was extracted from cells and analyzed for HCV RNA by quantitative real-time PCR. (B) Huh7.5 cells were co-cultured with free HCV virus or HCV exosomes with an MOI of 1 for 6 h after which viruses or exosomes were washed off and replaced with fresh complete culture medium. Cells were the cultured further or not over the time period indicated followed by treatment or not with Telaprevir (VX-950) at concentrations/time indicated. At indicated time points total RNA was extracted from cells and analyzed by Real time quantitative PCR for HCV RNA (C) Serum exosomes were isolated from three different patient groups (treatment naïve, treatment non-responders, and treatment responders) and co-cultured with primary human hepatocytes (PHH) for 8 h. Exosomes were then washed off and replaced with fresh medium for a further 40 hours. Culture supernatants obtained from PHH were analyzed for HCV RNA by quantitative real-time PCR. (D) Serum exosomes were isolated from HCV infected treatment naive patients using anti-CD63 immuno magnetic selection and co-cultured with primary human hepatocytes (MOI of 1 of infectious HCV viral particles or infectious HCV-exosome particles was used). Total RNA extracted from exosomes and PHH 48 h post infection and analysed by real-time quantitative PCR analysis for HCV RNA levels. Input exosome samples were appropriately matched by using 100 ng of total input exosome RNA and 100 ng of total infected cell RNA for HCV RNA comparison after infection to allow valid comparison of the starting exosome preparation. (E) Free HCV virus and CD63 selected HCV exosomes were isolated from HCV infected patient sera and used to infect primary human hepatocytes over the indicated time course (MOI = 1). Following infection cells were treated or not with Telaprevir (VX-950) at concentrations indicated for 24 h or not as indicated. Total RNA was then extracted from cells and analysed for HCV RNA using quantitaive real time PCR. Results are representative of exosomes from 4 different HCV infected treatment naive patients. Data are expressed as mean + SEM, p<0.05 was considered statistically significant by Mann-Whitney U test.

Observation of the presence of HCV RNA in exosomes prompted us to evaluate if exosomes from J6/JFH-1-infected hepatocytes or from HCV infected individuals could transmit infection to uninfected cells. We found that exosomes derived from supernatants of HCV J6/JFH-1 infected Huh7.5 cells mediated HCV infection after co-culture with uninfected Huh 7.5 cells (Fig. 4A and Figure S4) which could be inhibited by Telaprevir (VX-950) an NS3.4A serine protease inhibitor (Fig. 4B). Further, exposure of primary human hepatocytes (PHH) to exosomes isolated from treatment-naïve or treatment non-responder HCV infected patients resulted in effective virus infection and replication as indicated by detectable HCV RNA in the culture supernatants (Fig. 4C). Active virus replication after infection of PHH with HCV exosomes was indicated by a 2–3 log increase in HCV copy numbers in PHH at 48 hours after infection compared to the initial HCV copy numbers introduced by the HCV exosomes used for induction of infection (Fig. 4D). Additionally, the use of Telaprevir (VX-950), an NS3.4A serine protease inhibitor, could inhibit HCV replication caused by free virus and HCV exosomes in infected PHH (Fig. 4E).

### Exosomes mediate CD81, SB-RI and APOE receptor-independent transmission of HCV

**Figure 5:**
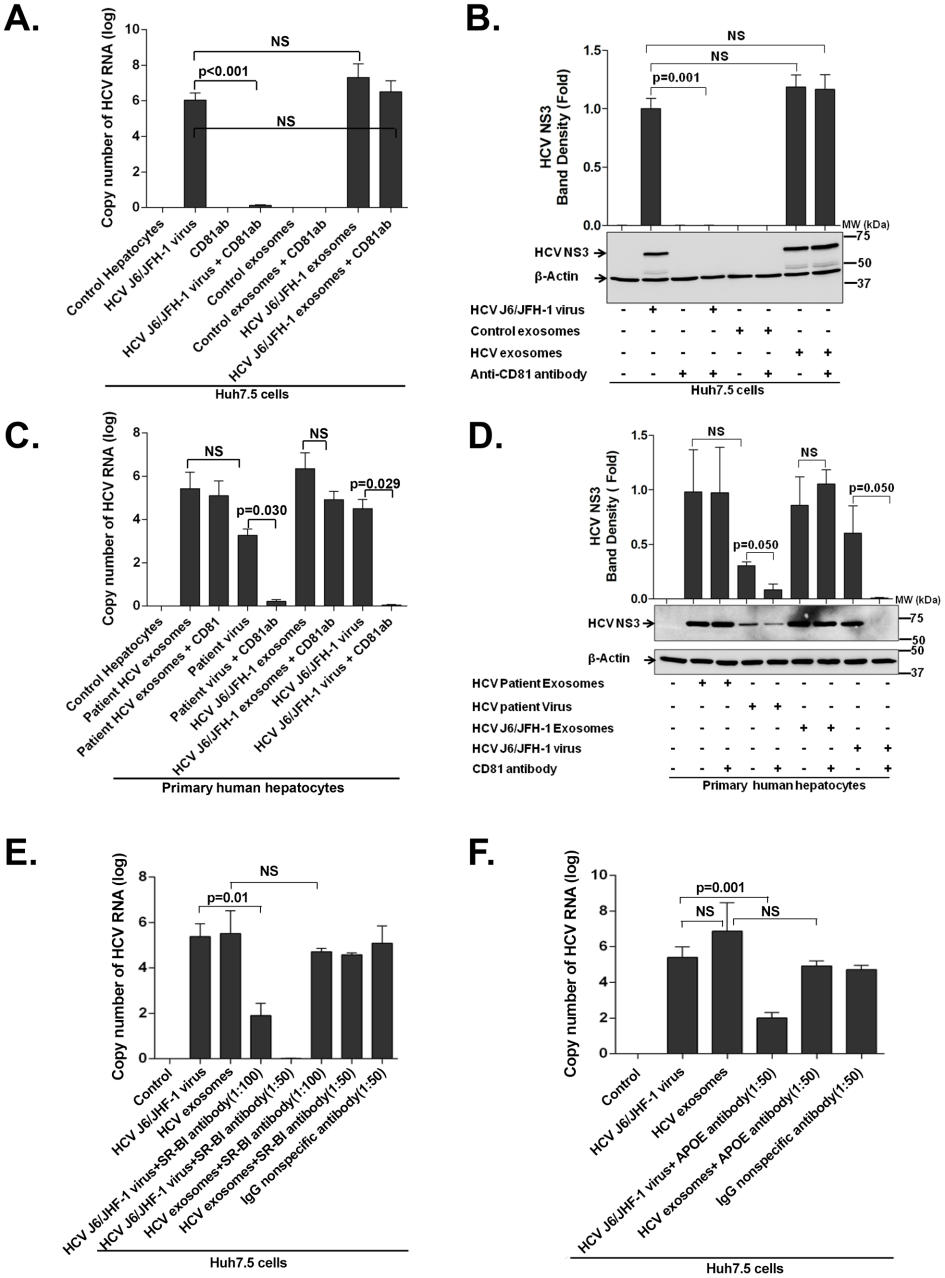
Exosomes from HCV J6/JFH-1 infected Huh7.5 cells and HCV patients mediate effective HCV transmission in the presence of anti-CD81, anti-SB-RI or anti-APOE antibody. (A&B) Huh7.5 cells were infected with HCV J6/JFH-1 virus or with exosomes from culture supernatants of HCV J6/JFH-1 infected Huh7.5 cells with CD81 antibody pre-treatment (1∶50 dilution) or not as indicated. Viruses and exosomes were washed off after 8 h of co-culture and fresh medium was added to cells followed by 40 h further culture at 37°C. Total RNA and protein was then extracted from cells and analyzed by (A) quantitative RT-PCR for HCV RNA and by (B) western blotting for HCV NS3 protein. (C & D) Primary human hepatocytes were infected with either serum exosomes or free HCV viruses from HCV treatment naïve patients; exosomes from Huh7.5-HCV J6/JFH-1 culture supernatants; HCV J6/JFH-1 virus along with anti-CD81 antibody pre-treatment for one hour before infection or not as indicated for 48 h. Total RNA and protein was then extracted from cells and analysed for HCV RNA by quantitative RT-PCR and by western blot for HCV NS3 protein. An MOI of 1 of infectious HCV virus and infectious HCV-exosomes were used for all infections. (E&F) Huh7.5 cells were infected with either HCV J6/JFH-1 exosomes or free HCV J6/JFH-1 viruses for 48 h after prior treatment of cells with anti-SB-RI antibody (1∶50 or 1∶100 dilution) (Fig. 5E) or anti-APOE antibody (1∶50 dilution) (Fig. 5F) at concentrations indicated for one hour before infections. Total RNA and protein was then extracted from cells and analysed for HCV RNA by quantitative RT-PCR and by western blot for HCV NS3 protein. An MOI of 1 of infectious HCV virus and infectious HCV-exosomes were used for all infections. Results presented are representative of 3 independent experiments expressed as mean + SEM, p<0.05 was considered statistically significant by Mann Whitney U test or ANOVA analysis for multiple comparisons performed with GraphPad Prism Version 5.0.

CD81, SB-RI, APOE and HCV E1/E2 proteins are important host and viral molecules for HCV infection [33]. We and others have shown that anti-CD81 and anti-HCV E1/E2 antibodies can block HCV infection [7], [12], [34]; however, in some instances antibody therapy in patients could not fully prevent HCV infection [16]. Based on these observations, we tested whether the presence of anti-CD81 antibodies would block exosome and cell free virus transmission of HCV. We found that anti-CD81 pre-treatment effectively blocked free HCV virus infection of target Huh7.5 cells, indicated by significantly low HCV RNA expression (Fig. 5A) and by lack of expression of HCV NS3 protein (Fig. 5B). However, exosomes containing HCV RNA could still transmit HCV infection despite anti-CD81 antibody pre-treatment (1∶50 dilution) (Fig. 5A & 5B). These findings were validated in primary human hepatocytes where anti-CD81 pre-treatment significantly inhibited free HCV virus infection but failed to prevent patient exosome-mediated HCV transmission (Fig. 5C & 5D). Additionally, SB-RI (Fig. 5E) or APOE (Fig. 5F) antibody pre-treatment could block HCV J6/JFH-1 free virus transmission but not HCV-exosome transmission of HCV to naïve Huh7.5 cells (Fig. 5E & 5F).

**Figure 6:**
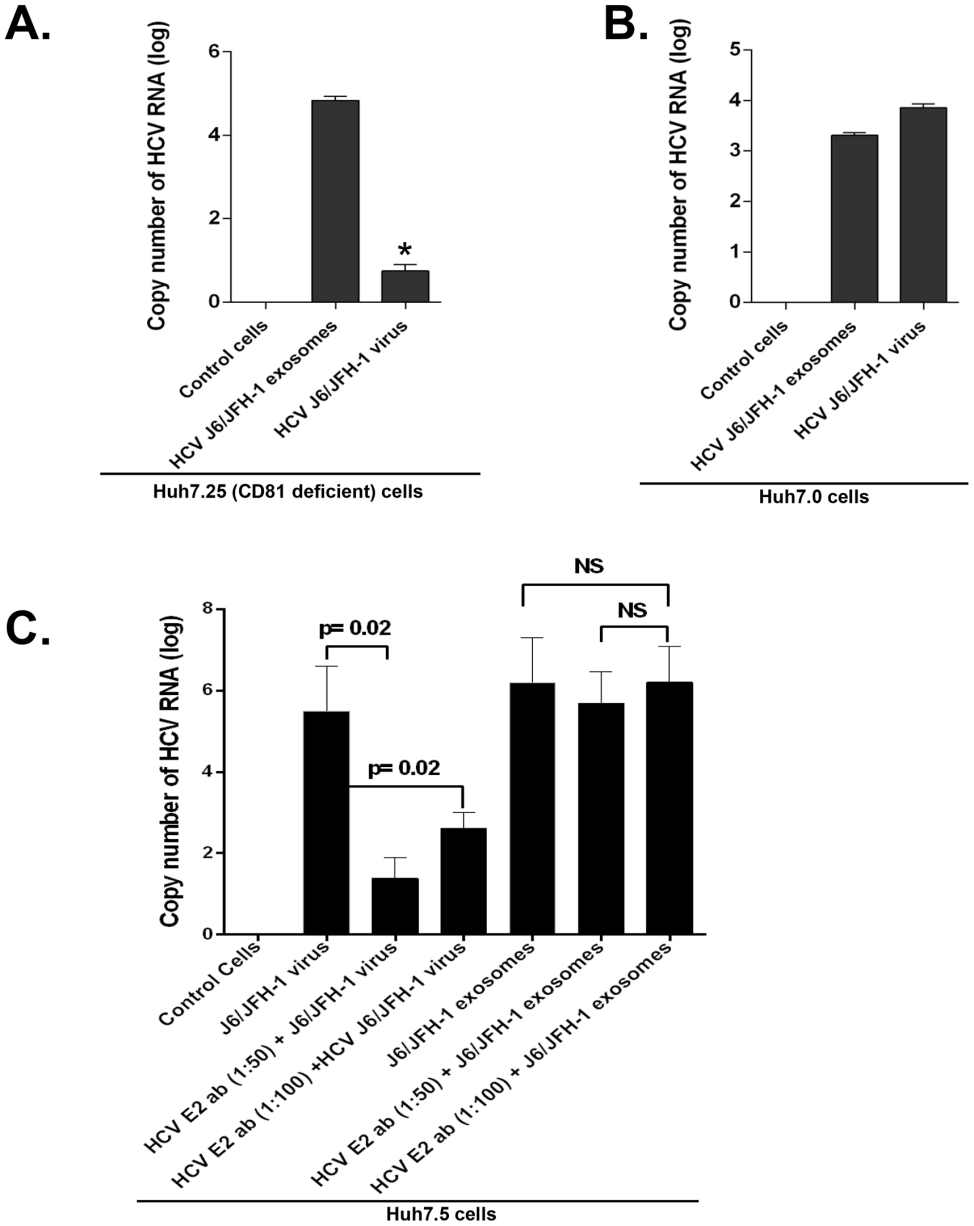
CD81 deficiency or anti-HCV E2 antibody treatment does not block effective HCV transmission by exosomes. (A) CD81 deficient Huh7.25 and (B) Huh7.0 cells were infected with HCV J6/JFH-1 virus or exosomes from culture supernatants of HCV J6/JFH-1 infected Huh7.5 cells as indicated. Viruses and exosomes were washed off after 8 h of co-culture and fresh medium added to the cells and cultured at 37°C for another 40 h. Total RNA was then extracted from cells and analysed for HCV RNA by RT-qPCR. An MOI of 1 of infectious HCV virus and infectious HCV-exosomes were used for all infections. Input exosome samples were appropriately matched by using 100 ng of total input exosome RNA and 100 ng of total infected cell RNA for HCV RNA comparison after infection to allow valid comparison. (C) Huh7.5 cells were infected with HCV J6/JFH-1 virus or with HCV exosomes from culture supernatants of HCV J6/JFH-1 infected Huh7.5 cells with anti-HCV E2 antibody pre-treatment (1∶50, 1∶100 dilutions) or not as indicated. Viruses and exosomes were washed off after 8 h of co-culture and fresh medium was added to cells followed by 40 h further culture at 37°C. Total RNA and protein was then extracted from cells and analyzed by quantitative RT-PCR for HCV RNA. Results presented are representative of 3 independent experiments expressed as mean + SEM, p<0.05 was considered statistically significant by Mann Whitney U test or ANOVA analysis for multiple comparisons performed with GraphPad Prism Version 5.0.

We next tested CD81-deficient Huh7.25-CD81 cells [35] and found that HCV exosomes could still mediate HCV transmission but infection rate with the free virus entry was significantly diminished (Fig. 6A). In the parental Huh7.0 cells, both exosomes and free HCV virus resulted in comparable extent of HCV infection (Fig. 6B). HCV E1 and E2 envelope glycoproteins which can modulate HCV infection [36] have been shown to associate with exosomes [23], thus we tested if anti-HCV E2 antibody treatment could block HCV transmission by exosomes. We found that anti-HCV E2 antibody treatment of HCV J6/JFH-1 virus could significantly block HCV transmission by free HCV particles but not by exosomes (Fig. 6C).

### Exosomes derived from HCV infected patient serum and Huh7.5-HCV J6/JFH-1 cells contain replication-competent HCV RNA in complex with Ago2-miR-122

**Figure 7:**
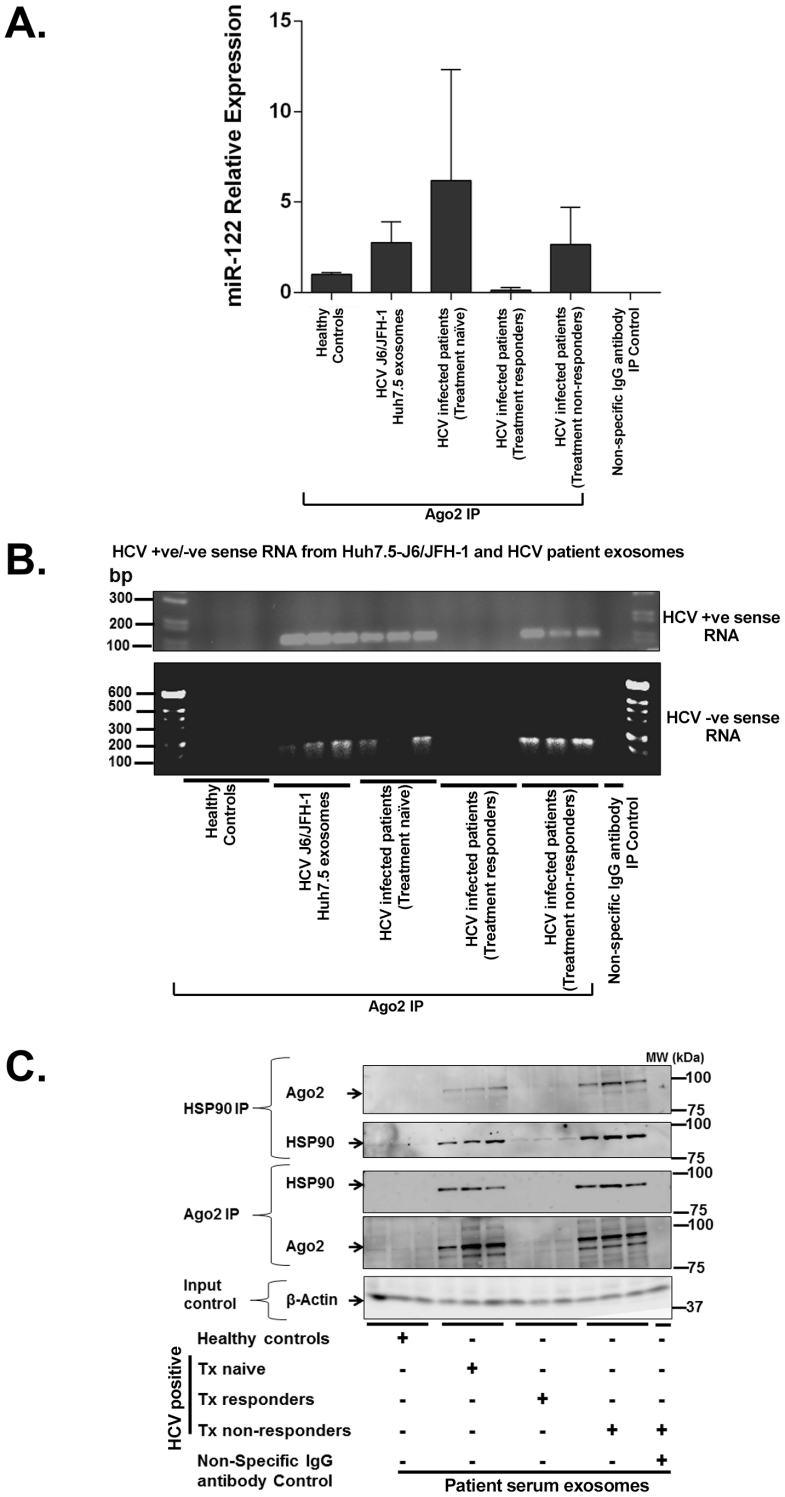
Exosomes from Huh7.5 cells and HCV infected patients' sera harbor replication competent HCV RNA and RISC complexes that enhance HCV infection. RNA ChIP analyses of Ago2 complexes in exosomes isolated either from culture supernatants of HCV J6/JFH-1 infected Huh7.5 cells or from patient samples were subjected to Ago2 pull down then total RNA isolation which was analyzed for (A) miR-122 expression, (B) for HCV positive (B-upper panel) and negative (B-lower panel) sense RNA by PCR. (C) Immunoprecipitation of Ago2 and HSP90 complexes from exosomal protein lysate isolated from culture supernatants of HCV J6/JFH-1 infected Huh7.5 cells or from patient serum samples. Normal rabbit IgG non-specific antibody was used as IP control antibody. Experiments are representative of 3 independent repeats for cell culture supernatants and patient samples (HCV patient genotypes 1a, 1b, 2a, and 3b).

Recent reports have demonstrated the role of Ago2 and miR-122 in enhancing HCV replication when bound to the 5′-UTR of HCV dsRNA [26]. We observed that the same MOI of free HCV viruses or HCV-exosomes resulted in a trend (but not statistically significant) of greater HCV transmission by exosomes compared to the cell free virus (Fig. 5). Based on this observation, we surmised that exosomes might contain replication-competent RNA in association with RISC complex proteins that could enhance HCV RNA stability and enhance viral replication [26], [37], [38]. Using RNA-chromatin immunoprecipitation (RNA-ChIP) analysis of exosomes isolated from HCV J6/JFH-1 infected Huh 7.5 cells or HCV infected patients after Ago2 pull-down, we found that Ago2 was associated with miR-122 (Fig. 7A), positive sense HCV RNA (Fig. 7B upper panel) and, in some cases, negative sense HCV RNA (Fig. 7B lower panel). Using free HCV virus RNA and RNA from HCV infected cells we confirmed primer specificity for detection of positive and negative sense HCV RNA (Figures S5A & S5B). Additionally, using co-immuno precipitation, we confirmed that HSP90 and Ago2 formed complexes within the HCV containing exosomes likely providing further stabilization of the HCV RNA-replication complex (Fig. 7C) [39].

These striking observations indicate that serum exosomes from some HCV infected treatment-naïve patients contain positive sense RNA of HCV virus and are able to transmit active HCV infection. We found that, even in the few patients where we could not detect viral RNA in the exosomes due to the limitation of the sensitivity of the Real Time PCR method (Table 1 & 2), HCV infection of PHH was still evident (Fig. 4C). Furthermore, replication competent, negative sense HCV RNA was also present in some treatment-naïve and in all non-responder patients (Table 1 & 2).

**Table 1:** Clinical parameters of HCV patients used for serum miRNA isolation.

Parameters	Distribution
Gender: Male/female	30/28
Age	54.07 ± 1.92
Viral load (IU/mL)	5×10^4^ – 9.3×10^6^ (HCV patients only)
Genotype	1a, 1b, 2b, 3a (HCV patients only)
Patient numbers in each Category	Healthy Control: 12
	HCV treatment responders:6
	HCV treatment responders:6
	HCV Treatment non responders: 6

**Table 2:** HCV RNA content of patient exosomes.

HCV RNA content of patient exosomes	Positive sense HCV RNA	Negative sense HCV RNA
Control patients	None	None
HCV infected treatment Naive	14 out of 34	7 out of 34 (all 7 had positive sense HCV RNA)
HCV treatment Responders	0 out of 6	0 out of 6
HCV Treatment non-responders	6 out of 6	6 out of 6

### Inhibition of miR-122 or HSP90 blocks exosome-mediated transmission of HCV in Huh7.5 cells

**Figure 8:**
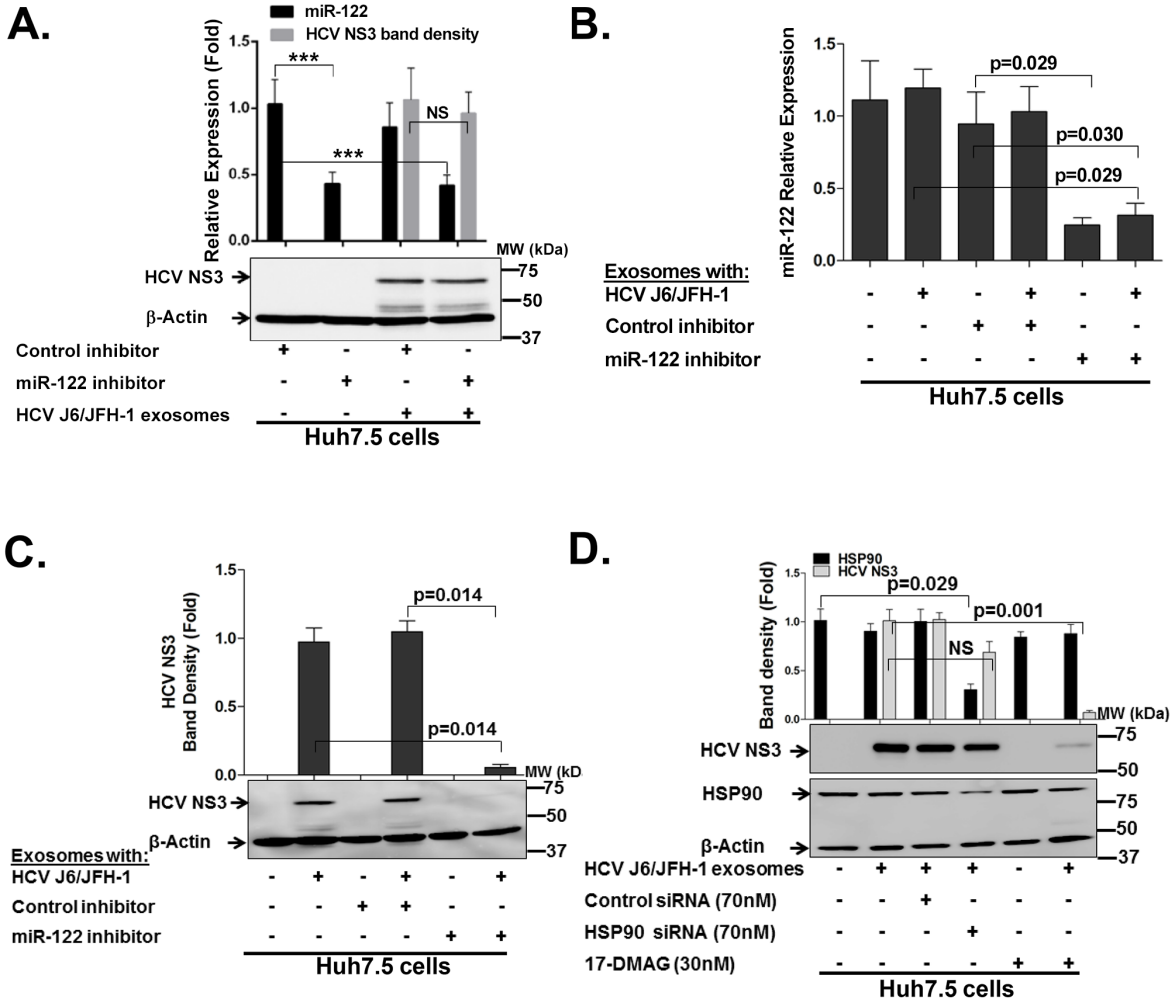
HCV transmission by exosomes can be blocked by miR-122 or HSP90 functional inhibition within exosomes. (A) miR-122 inhibitor or control inhibitor complexed with Altogen liver specific in-vivo delivery reagent was co-cultured with Huh7.5 derived control exosomes for 20 mins and exosomes re-purified to exclude carryover of transfection reagent. The derived exosome containing miR-122 inhibitor or control inhibitor was co-cultured with Huh7.5 cells for 12 h. Post miR-122 inhibition or not using loaded exosomes for delivery, cells were washed and infected with HCV exosomes as indicated (MOI of 1 of infectious HCV-exosome particles was used) for 24 h. Total RNA and proteins was then extracted from cells and analyzed for miR-122 by RT-qPCR and western blot analysis for HCV NS3 with B-actin serving as a loading control. (B) miR-122 inhibitor or control inhibitor complexed with Altogen liver specific in-vivo deliver reagent was co-cultured with HCV exosomes for 20 mins and exosomes re-purified. HCV exosomes loaded with miR-122 inhibitor or not as indicated were then transferred unto Huh7.5 cells and co-cultured for 24 h in complete tissue culture medium. Following HCV exosome infection co-cultures as indicated, total protein was extracted from Huh7.5 cells and analyzed for (C) HCV NS3 protein. (D) Western blot analyses of HCV NS3 and HSP90 proteins 48 h after HSP90 siRNA or DMAG pre-treatment (1 h) and 24 h after co-culture with exosomes from HCV J6/JFH-1 infected Huh7.5 cell culture supernatants. An MOI of 1 was used for all HCV exosome infections. Data presented here is representative of 3–4 independent experiments with p<0.05 considered to be statistically significant by Mann Whitney U test or ANOVA for multiple comparison performed with GraphPad Prism Version 5.0.

Given that exosomes from HCV-infected treatment-naïve and treatment non-responders contained Ago2 in complex with miR-122, and HSP90, we tested the effect of miR-122 or HSP90 inhibitors which have been suggested for HCV treatment [29], [37], [40]. Delivery of a miR-122 inhibitor resulted in about 50% reduction in miR-122 levels in Huh7.5 cells that is significant considering the high abundance of miR-122 in hepatocytes (Fig. 8A). However, inhibition of miR-122 in Huh7.5 cells prior to infection with HCV exosome failed to significantly suppress HCV transmission (Fig. 8A). Given that exosomes harbored HCV in complex with miR-122/HSP90, we hypothesized that miR-122 in the exosomes provides advantages for HCV transmission. To test this hypothesis, we transfected HCV-exosomes with a miR-122 inhibitor or control, washed and re-purified the miR-122 inhibitor- or control inhibitor-loaded HCV-exosomes and used them for infection of naïve Huh7.5 cells. The miR-122 inhibitor-loaded HCV-exosomes resulted in a significant reduction in intracellular miR-122 levels in Huh7.5 cells (Fig. 8B). Importantly, we found reduced virus transmission by HCV-exosomes loaded with the miR-122 inhibitor as indicated by decreased HCV NS3 protein compared to the controls (Fig. 8C).

We also assessed the potential of the HSP90 activity inhibitor, 17-DMAG, or HSP90 siRNA treatment to modulate HCV infection transmitted by exosomes (Fig. 8D). We found that DMAG treatment but not HSP90 siRNA treatment could significantly block exosome-mediated HCV transmission (Fig. 8D).

### Vacuolar-type H+-ATPase inhibitor and proton pump inhibitor can block exosome and free virus transmission of HCV to Huh7.5 cells

**Figure 9:**
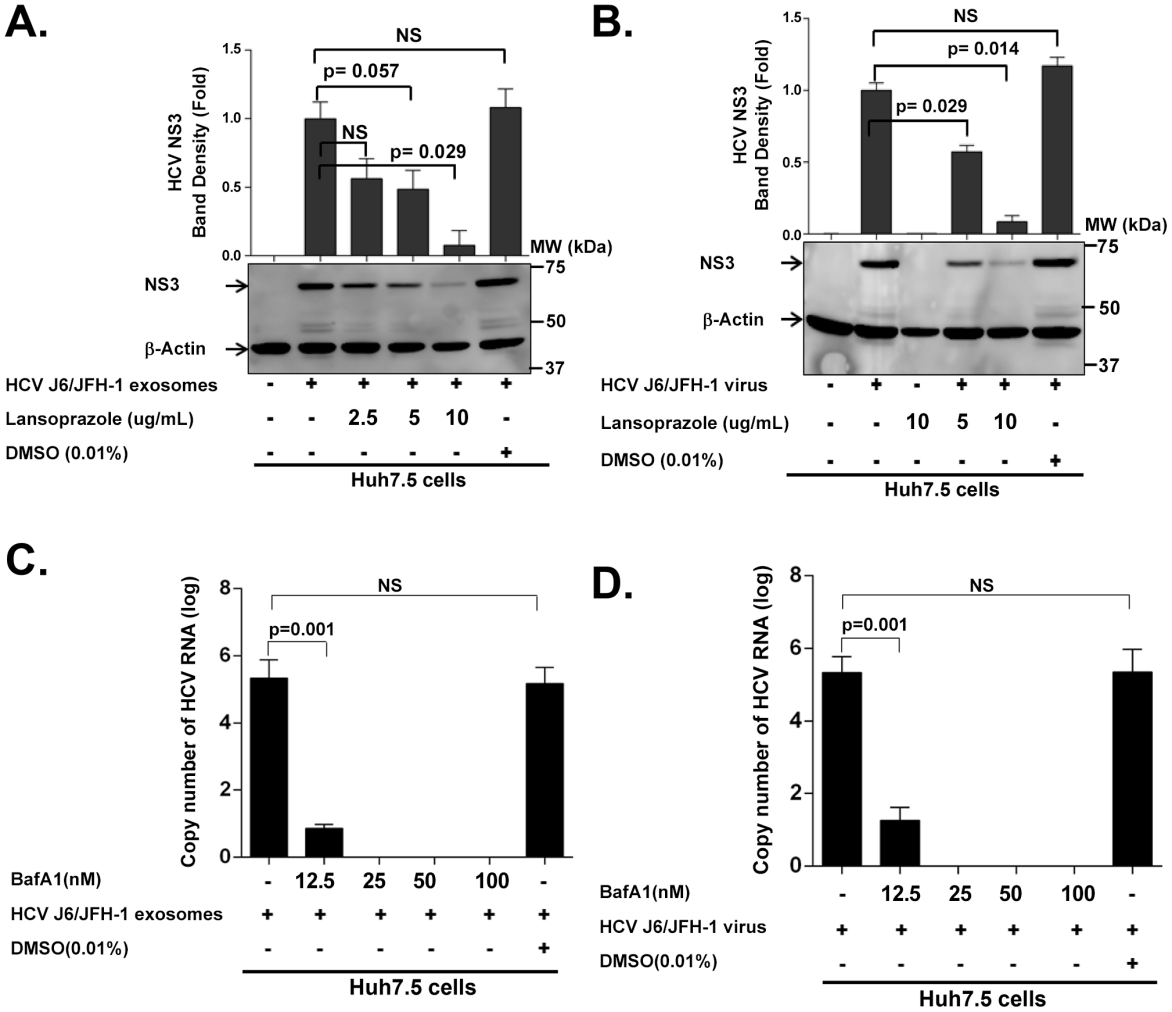
HCV transmission by exosomes and free virus can be blocked by proton pump inhibitor (Lansoprazole) and Vacuolar-type H+-ATPase inhibitor (bafilomycin A1). (A&B) Huh 7.5 cells were pre-treated with Lansoprazole (2.5 µg/ml, 5 µg/ml, and 10 µg/ml) for 1 h at indicated concentrations, and then infected with (A) HCV exosomes or (B) cell free HCV virus for 24 h. Total protein was then extracted from cells and analyzed for HCV NS3 protein. (C&D) Huh 7.5 cells were pre-treated with bafilomycin A1(Baf A1) (12.5 nM, 25 nM, 50 nM, and 100 nM) for one hour at concentrations indicated, then infected with (C) HCV J6/JFH-1 exosomes or (D) cell free HCV virus for 24 h. Total RNA was then extracted from cells and analyzed for HCV RNA. An MOI of 1 of HCV-exosomes and cell free HCV virus were used for all infections. Data presented here is representative of 3 independent experiments with p<0.05 considered to be statistically significant by Mann Whitney U test.

Previous data showed that viral HCV entry and subsequent infection can be prevented by administering vacuolar-type H+-ATPase inhibitor [41]. Moreover, Meertens et al [42] reported that entry of HCV pseudoparticles (HCVpp) was efficiently blocked by bafilomycin A1, a specific vacuolar-type H+-ATPase inhibitor, which neutralizes the pH in early endosomes and injures progression of endocytosis beyond this level. Exosome entry through endocytosis is reported to be pH dependent in the traffic of tumor exosomes in regulating both their release and uptake by tumor cells [43]. Based on these reports, we set up an *in vitro* model utilizing a vacuolar-type H+-ATPase inhibitor (bafilomycin A1) or a proton pump inhibitor (Lansoprazole) to study the role of low pH in favoring HCV infected exosome uptake in Huh 7.5 cells. We found that low pH plays a role in the entry of infected exosome into the Huh 7.5 cells and infection by HCV infected exosomes can be blocked using vacuolar-type H+-ATPase or a proton pump inhibitor. Our data show that both Lansoprazole (Fig. 9A & 9B) and bafilomycin A1 (Fig. 9C & 9D) could significantly inhibit HCV transmission by exosomes and cell free HCV viruses to Huh7.5 hepatoma cells in a dose-dependent manner without causing significant cellular cytotoxicity (Figures S6A & S6B).

## Discussion

Exosomes are found in different biofluids and represent a small (40–150 nm) subpopulation of extracellular vesicles of endocytic origin released by almost all cell types. They act as natural carriers of genetic materials, namely miRNA, mRNA and proteins [44], [45]. Notably, exosomes have been shown to mediate disease transmission caused by bacteria, infectious prion protein, and viruses [46], [47]. In the context of HCV, recent studies showed that hepatocyte-derived exosomes containing viral RNA induced production of IFN-*α* in plasmacytoid dendritic cells (pDCs) in vitro [24]. In this study, we demonstrate that circulating exosomes derived from sera of treatment-naïve HCV infected individuals or HCV treatment non-responder individuals contain HCV virus that can transmit active HCV infection to primary human hepatocytes, confirmed with the observation of a 2–3 log increase in HCV copy numbers in PHH compared to the initial HCV copy numbers in exosomes used for infection indicated virus replication. A recent study also reported exosome-mediated transmission of HCV in Huh7.5 cells [48]. Our observations confirmed and extended a recent report that also found that exosomes derived from HCV J6/JFH-1 infected Huh7.5 cells can shuttle virus to normal Huh7.5 cells and establish a productive infection. Using a stringent isolation methodology of serial filtration followed by density separation and immune magnetic CD63-positive exosome isolation, we optimized a method of HCV exosome isolation without carryover of free virus thereby further underscoring the capacity of exosomes to transmit HCV infection.

Our findings showed for the first time that exosomes from sera of HCV infected patients or culture supernatants of HCV J6/JFH-1 infected Huh7.5 cells can mediate effective CD81, SB-RI, HCV E2 and APOE -independent HCV transmission to hepatocytes. A recent report by Ramakrishnaiah et al [48] indicated that exosomes can mediate partial CD81-independent HCV transmission in Huh7.5 cells, however in that study cell free HCV transmission could not be fully excluded. Our results indicate that exosomes that are devoid of free virus contamination are capable of HCV transmission even in the presence of a potent anti-CD81, anti-SB-RI, anti-HCV E2 and anti-APOE antibody treatment and in CD81-deficient cells. These observations could explain in part why neutralizing antibodies or therapies that target host/viral protein interactions at the level of cell entry can be compromised and likely occur via cell-to-cell transmission by exosomes.

Given that infections with HCV-exosomes compared to the same MOI of free HCV virus particles, showed a tendency for higher levels of HCV transmission to hepatocytes, it was unclear if these exosomes contained replication competent HCV RNA, factors that enhanced virus replication or facilitated mechanisms of exosomes entry to target cells. Recently, reports have consistently demonstrated that RISC-like complexes involving Ago2 and miR-122 can protect the HCV 5′ internal ribosome entry site (5′ IRES) and enhance HCV replication [26], [38]. We found higher miR-122 expression in HCV J6/JFH-1 infected Huh7.5 cells derived exosomes compared to HCV infected patient exosomes and their respective controls, possibly as a result of suppressed interferon production in Huh7.5 cells since Huh7.5 cells harbor a mutation in the dsRNA sensor retinoic acid-inducible gene-I (RIG-I) [49]–[51]. However, using RNA ChIP analyses we found that exosomes from HCV J6/JFH-1 infected Huh 7.5 cells and exosomes from the two patient groups that have active infection, treatment-naïve and treatment non-responders, showed increased proportion of miRNA-122 in complex with Ago2. Additionally, it was remarkable that Ago2 and miR-122 bound to the HCV 5′-UTR was also in association with HSP90 which has been shown to stabilize RISC complexes [52] and potentially increase HCV replication. Our observations support a hypothesis whereby exosomes mediate higher HCV transmission because they contain replication-competent viral RNA, as well as, known HCV replication enhancers- Ago2 [26], miR-122 [26], [29], [37], and HSP90 [37], [53], [54]. Additionally, HCV RNA in exosomes might mediate higher levels of infection possible due to the higher stability of HCV RNA when associated with Ago2 and miR-122 as suggested by Shimakami et al [39].

The presence of host proteins within HCV-exosomes is a clever strategy by the virus to ensure effective replication once in the endoplasmic reticulum (ER) given that the ER does not contain these exosomal proteins [55]. Our novel findings may translate and offer possible clinical implications to HCV treatment resistance with interferon/ribavirin given that exosomes from HCV-infected treatment resistant patients contained HCV negative sense RNA, which is mostly associated with replication-competent HCV RNA. Strikingly, only some of the treatment-naïve patients with HCV positive-sense RNA detected in their exosomes contained negative-sense HCV RNA (Table 2). Importantly, none of HCV treatment responder patients harbored detectable HCV RNA in their serum-derived exosomes consistent with their status of HCV viral clearance. The implication of our findings needs additional clinical follow-up to determine whether treatment and/or disease outcome using anti-HCV immune therapies would be influenced by the composition of serum-derived exosomes in HCV infected patients.

Based on our novel findings that exosomes can mediate virus transmission via CD81, SB-RI and APOE -independent mechanisms potentially compromising the efficacy of HCV immunotherapies, we next aimed to test therapeutic alternatives. We analyzed the potential use of miR-122 inhibition and DMAG treatment both of which have been successfully explored for HCV treatment but not yet assessed in the context of exosome-mediated HCV transmission. We found that using an exosome targeted miR-122 inhibitor system or the HSP90 inhibitor, DMAG, which could inhibit the effective function of these host factors which modulate HCV infection/replication, could significantly suppress HCV transmission by exosomes. Strikingly, attenuation of HSP90 or miR-122 levels by siRNA knockdown and miR-122 inhibitor in target cells was not sufficient to inhibit HCV transmission via exosomes. This could be due to the fact that HCV exosomes contain all the necessary viral and host protein factors that are otherwise not present in the endoplasmic reticulum [18], and can thus mediate effective replication once cellular entry is accomplished by exosome uptake.

Since exosomes originate from lumen of multivesicular bodies (MVBs), their release and uptake are associated with the endocytic pathway [56]. Acidification of intracellular organelles is reported to be fundamental to the function of the endocytic pathways and exosomes uptake [57]. The vacuolar H^+^-ATPases (V-ATPases) and proton pumps are responsible for generating and maintaining intra-cellular pH gradients across cell membranes. Disruption to their functions were reported to be accompanied by lysosomal dysfunction and impaired endocytosis [58], [59]. From another perspective, several reports show a crucial role of low pH and endosome acidification for triggering virus entry, not addressing the distinction between exosomes and cell free viruses [41], [60]. Here we show that the use of bafilomycin A1, a specific vacuolar H+-ATPase proton pump inhibitor, and Lansoprazole, a proton pump inhibitor, prevented the capacity of exosomes and cell free virus to transmit infection, suggesting their use in the treatment regimens for HCV infection. This usage seems to be more influential as it is reported that the intracellular pH was not noticeably changed by dosages less than 100 nM of bafilomycin A1 and for a short period of time (4 h), which is reported to be the critical time point for effect of bafilomycin A1 to prevent viral entry [61].

**Figure 10:**
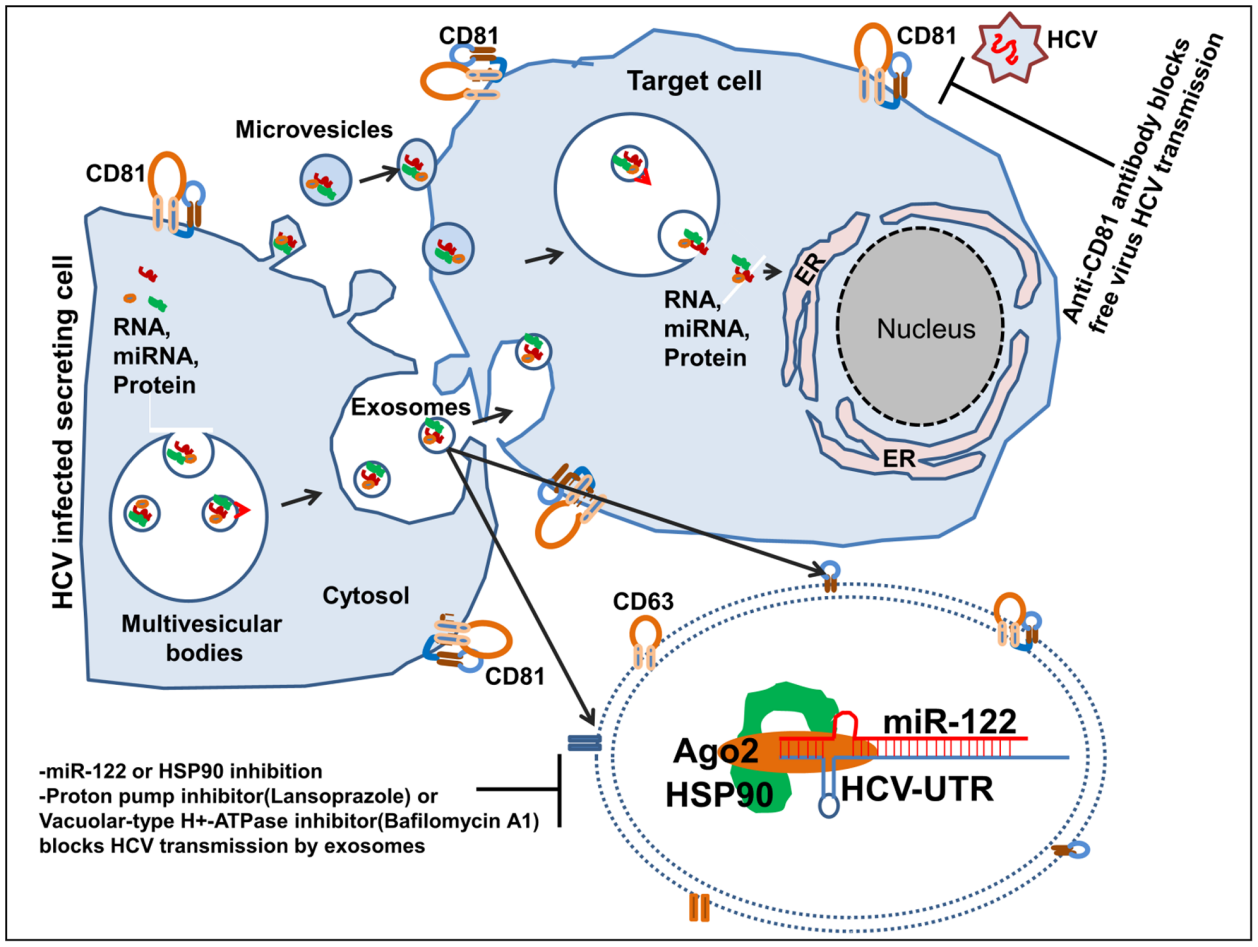
Schematics of exosome mediated HCV transmission and possible therpaeutic strategies. HCV infected hepatocytes form multivesicular bodies (MVB) by taking up bits of the cytoplasm and its contents (proteins [HSP90], miRNA [miR-122] and RNAs [HCV RNA]) into membrane-bound vesicles. These MVB in HCV infected hepatocytes then fuse with the plasma membrane and release their contents, including numerous small vesicles (exosomes), outside the cell. Exosomes released from HCV infected hepatocytes contain replication competent HCV RNA in complex with miR-122, Ago2, and HSP90. Further, exosomes released from HCV infected hepatocytes are capable of CD81-independent HCV transmission to a naïve hepatocyte. The use of HSP90, miR-122, proton pump and Vacuolar-type H+-ATPase inhibitors can significantly limit the capacity of HCV transmission by exosomes.

In summary, our novel findings, illustrated in Figure 10 provide mechanistic insights into how exosomes can mediate indirect cell-to-cell viral receptor independent transmission of HCV. Furthermore, we provide evidence that circulating exosomes of HCV infected patients can infect primary human hepatocytes. Additionally, our findings further support the rationale for using miR-122 inhibitors, HSP90 inhibitor, and potentially proton-pump and Vacuolar-type H+-ATPase inhibitors to prevent exosome-mediated HCV transmission.

## Materials and Methods

### Cell lines, primary human hepatocytes (PHH) and HCV J6/JFH-1 virus

Huh7.5, Huh7.0 (a gift from Dr. Charlie Rice, Rockefeller University, New York) and CD81-deficient Huh7.25 [a gift from Dr. Takaji Wakita (National Institute of Infectious Disease, Tokyo, Japan) and Dr. T. Jake Liang (NIDDK, National Institutes of Health, USA)] cells were cultured as previously described [35], [49] with slight modification, using exosome depleted FBS (System Bioscience cat. #EXO-FBS-50A-1). Primary human hepatocytes were obtained from the National Institutes of Health (NIH) liver tissue cell distribution system (LTCDS; Minneapolis, MN, USA; Pittsburgh, PA; Richmond, VA, USA), which was funded by NIH contract #N01-DK-7-004/HHSN2670070004C and from BD Bioscience. Highly infectious and replication competent HCV J6/JFH-1 virus (genotype 2a) were generated as previously described [62]. The pFL-J6/JFH-1 plasmid used for virus generation was provided by Dr. Charlie Rice and Dr. Takaji Wakita (National Institute of Infectious Disease, Tokyo, Japan). HCV J6/JFH-1 virus concentration in culture supernatants was determined using NanoSight LM10 (MOI of infectious viral particles or infectious exosomes) and by quantitative real-time PCR as previously described [37].

### Ethics statement

Subjects were recruited from the Hepatology clinic at the University of Massachusetts Medical School. This research protocol was reviewed by the Committee for the Protection of Human Subjects in Research at the University of Massachusetts Medical School (IRB #2284). All subjects who donated samples for this project provided signed written informed consent. Subjects were assessed for baseline demographics, Hepatitis C viral serology and liver function parameters (Table 1). Healthy control subjects had no evidence of systemic disease, HCV infection, or other liver diseases. Informed consent was obtained from all subjects. Blood samples were drawn and serum samples were analyzed for HCV RNA using RT-PCR and processed as subsequently indicated.

### Exosome isolation and purification from cell lines and patient samples

Huh7.5 cells and HCV J6/JFH-1 infected Huh7.5 cells were maintained in DMEM low glucose medium supplemented with 10% exosome depleted FBS and 1% penicillin/streptomycin (Gibco, cat. #15140-163). Cell culture supernatants following cell infection or not, or patient serum samples were collected, centrifuged at 2500 rpm for 10 mins at 4°C to remove cell debris, then filtered through a 0.2 µm filter. The 40 mL of filtered culture supernatant for exosome isolation was concentrated to a final 1 mL volume using the Amicon Ultra-15 Centrifugal Filter Unit with Ultracel-100 membrane (Millipore, cat. #UFC910024). Concentrated culture supernatants or filtered patient serum (500 uL) were mixed with the appropriate volume of Exoquick-TC reagent (System Biosciences cat. #EXOTC10A-1) or Exoquick (System Bioscience cat. #EXOQ5A-1) respectively, for exosome isolation according to the manufacturers' specification. Samples were gently mixed and incubated for 1 h at 4°C. Following incubation, exosomes were precipitated by centrifugation at 1400 rpm for 10 mins at 4°C. The recovered exosomes were re-suspended in 1× phosphate buffered saline (PBS). Positive selection of exosomes was done using anti-CD63 immuno-magnetic capturing with primary anti-CD63 antibody (Abcam cat. #ab8219 and Santa Cruz cat. #15363) followed by corresponding secondary antibody coupled to magnetic beads (Miltenyi Biotec cat. #130-048-602). The Miltenyi Biotec MidiMACS separator was used with LD columns (cat. # 130-042-901) for exosome isolation.

### Electron microscopy

Exosomes isolated by positive anti-CD63 immuno-magnetic bead selection were re-suspended in PBS and transferred to a formvar-coated copper grid then allowed to settle/attach for 30 minutes. The grid was washed by sequentially positioning droplets of PBS on top and using absorbing paper in between. The samples were then fixed by drop-wise addition of 2% paraformaldehyde onto parafilm and placing the grid on top of the paraformaldehyde drop for 10 min. Fixation was followed by five washes with deionized water and samples contrasted by adding 2% uranyl acetate for 15 minutes. Afterward, the samples were embedded by adding a drop of 0.13% methyl cellulose and 0.4% uranyl acetate for 10 minutes. The grid was visualized using a Philips CM10 transmission electron microscope and images were captured using a Gatan CCD digital camera.

### Quantification of HCV-exosomes, purified HCV infectious virus particles and HCV RNA copy numbers

Quantification of immuno-magnetic CD63 bead captured infectious HCV J6/JFH-1-exosomes and HCV J6/JFH-1 virus preparations was determined using NanoSight LM10 system (NanoSight, Amesbury, UK) equipped with a fast video capture and Nanoparticle Tracking Analysis (NTA) system, according to the manufacturer's instructions. Quantification of HCV RNA copy numbers was done as previously described [31].

### siRNA and miRNA inhibitor constructs and cell transfections

The following siRNA and miRNA inhibitors were used: human HSP90 siRNA (Santa Cruz cat. #sc-35608); control siRNA (Santa Cruz cat. #sc-44236), hsa-miR-122 anti-miR miRNA Inhibitor (Ambion, Austin, Tx cat. #AM11012) and anti-miR Negative Control (Ambion, Austin, Tx cat. #AM17010). miRNA or control inhibitors were complexed with the liver specific in vivo Altogen delivery reagent (Altogen Biosystems cat. #5060) which was loaded into control exosomes or HCV exosomes then co-cultured with target cells as indicated. Specific SiRNA or control siRNA was complexed with FugeneHD (Roche cat. # 04709705001) and transfected into target cells according to the manufacturer's specifications as indicated.

### RNA Chromatin immunoprecipitation (ChIP) and co-immunoprecipitation analysis of exosome samples

Exosomes isolated from cell culture supernatants or patient serum samples were fixed at room temperature with 4% formaldehyde buffered saline. Afterward, exosomes were lysed in SDS ChIP lysis buffer (Millipore cat. # 20-163) supplemented with protease inhibitor and RNase inhibitor. Total exosome proteins were pre-cleared with protein G beads. 50 µg of total protein was incubated with Ago2 antibody. Immunoprecipitation was performed for 90 minutes at 4°C using 10 µg/ml primary Ago2 antibody and normal rabbit IgG (Santa Cruz cat # sc-2027) non-specific antibody used as IP control. A mixture of Protein A/G PLUS-Agarose beads (Santa Cruz cat. #sc-2003) was added, and the incubation was continued for an additional 60 minutes. The samples were washed with SDS ChIP lysis buffer supplemented with protease inhibitor and RNase inhibitor. The immunoprecipitated protein-RNA complex was either used for Western blot analysis or RNA purification after Ago2 pull down using the Zymo research Direct-zol RNA MiniPrep kit (cat. #R2050), according to the manufacturer's specification. TaqMan MicroRNA assay was used for quantification of miRNA, using a CFX Connect Real-Time PCR Detection System (Philadelphia, USA). The exosome miRNA data was normalized to Cel39 and fold change was calculated using delta-delta ct method as previously described [63].

### Western blot analysis

Western blots were performed using the following established protocols. Briefly, proteins were resolved on 10% SDS-PAGE gels. After electrophoresis resolved proteins were transferred onto nitrocellulose membranes. Following protein transfer, membranes were blocked for 1 hour in PBS containing 5% non-fat dry milk and 0.1% Tween-20. Blots were then incubated overnight with primary antibody at 4°C. The following primary antibodies were used: anti-HCV NS3 (Abcam cat. #ab13830); anti-HSP90 (Cell Signaling cat. #4874); anti-CD63 (Abcam cat. #ab8219 used for western blotting and Santa Cruz Biotechnology cat. #sc-15363 used for exosomes purification); anti-Ago2 (Sigma cat. #SAB4200274); anti-CD81 (Santa Cruz Biotechnology cat. #sc-23962), normal rabbit IgG-AC antibody (Santa Cruz Biotechnology cat. # sc-2345); anti-beta actin [Ac-15] (Abcam, cat. #ab6276). The membranes were then incubated for 1 hour with horseradish peroxidase-conjugated secondary antibodies (dilution 1∶10,000) that included: goat anti-mouse IgG-HRP (Santa Cruz Biotechnology cat. #sc-2005); goat anti-rabbit IgG-HRP (Santa Cruz Biotechnology cat. #sc-2004). Finally, the proteins were visualized with the Clarity Western ECL substrate (BioRad, cat. #170-5061) chemiluminescence system according to the manufacturer's protocol using the Fujifilm LAS-4000 luminescent image analyzer.

### Quantification of miRNA or HCV RNA expression in exosomes and cell lines

Prior to total RNA isolation, equal volume of plasma (500 µL) or 500 uL of 10 mL concentrated culture supernatant samples were thawed on ice, mixed with QIAzole (Qiagen), vortexed and incubated at RT for 5 mins. Synthetic C. elegans (cel)-miR-39 was spiked and after this step total RNA was extracted using Zymo research Direct-zol RNA MiniPrepKit as per instructions. TaqMan miRNA Assay (Applied Biosystems) was used to analyze the miRNA from serum or plasma samples. Cel-miR-39 was used to normalize the technical variation between the exosomes samples and when comparing miRNA or HCV RNA content in cell lines compared to exosomes. Quantification of miR-122 was performed using Taqman microRNA assays (Applied Biosystems). RNU48 was used as an endogenous control for miR-122 expression in cells and Cel-miR-39 was used as an exogenous control to normalize for technical variation in RNA isolation for determining miR-122 levels in exosomes.

### Analysis of HCV positive and negative sense RNA

After RNA isolation as indicated, reverse transcription was performed by two different methods both of which were designed to amplify the 5′-UTR of HCV as previously described [37]. Briefly, positive sense RNA was amplified involving a first cDNA synthesis reaction using 500 ng of total RNA using the Bio-Rad cDNA synthesis kit according to the manufacturer's specification. The positive sense HCV 5′ UTR was then amplified using the following primer sequence: HCV Forward Primer: 5′-TCTGCGGAACCGGTGAGTAC-3′; HCV Reverse primer: 5′-TCAGGCAGTACCACAAGGCC-3′. HCV negative sense RNA was detected using primers and PCR conditions as previously described [64].

### Vacuolar-type H+-ATPase inhibitor (bafilomycin A1), proton pump inhibitor (Lansoprazole) and for inhibition of HCV Huh 7.5 cells derived exosomes and virus entry/replication

Bafilomycin A1 was purchased from Sigma Aldrich and the proton pump inhibitor; Lansoprazole (Prevacid 24 hr OTC, Novartis), was purchased over the counter. Lansoprazole was dissolved in DMSO and applied to Huh7.5 cells at concentrations of 2.5 µg/ml, 5 µg/ml, and 10 µg/ml. Telaprevir (VX-950) was purchased from Selleckchem and used as previously described [65]. One hour later, HCV virus suspension and HCV infected exosomes (captured with CD63) were added to the cells. Twenty-four hour later, the cells were washed 3 times and assessed for viral structural protein, NS3. Bafilomycin A1 was dissolved in DMSO and applied to the Huh 7.5 cells at concentrations of 12.5 nM, 25 nM, 50 nM, and 100 nM, while the concentration of DMSO in the final treatments was 0.01%. One hour later, HCV virus suspension and HCV infected exosomes (captured with anti-CD63 antibody) were added to the cells. After 24 h, the cells were washed 3 times and assessed for viral RNA entry.

### Lactate dehydrogenase (LDH) assay

The LDH toxicity assay kit (Abcam Cat. # ab65393) was used according to the manufacturer's specification. Briefly, released LDH in culture supernatants of Huh7.5 cells after 24 h co-culture with different concentration of Bafilomycin A1 and Lansoprazole was measured as the indicator of lysed cells. The percentage of cytotoxicity was measured by subtracting LDH content in remaining viable cells from total LDH in untreated controls. Staurosporine (20 nM) (Abcam, Cambridge, MA) treatment of Huh7.5 cells for 12 h was used as positive control. The final absorbance was measured at 490 nm. All experiments were performed in triplicate.

### Blocking antibody experiments

Cells, as indicated, were treated with blocking antibodies to target HCV host receptors for one hour prior to infection with either HCV exosomes, HCV J6/JFH-1 virus or not as indicated. Blocking antibodies used included: anti-CD81 antibody (Santa Cruz Biotechnology cat. # sc-23962), anti-Scavenging Receptor (SR-BI) antibody (Abcam, cat. # ab52629), anti-HCV E2 antibody (GeneTex cat. # GTX103353) and anti-ApoE antibody (Millipore Cat. #: AB947).

### Enzyme-Linked Immunosorbent Assay (ELISA) for APOE and APOB

Culture supernatants of Huh7.5 cells infected or non-infected with HCV (J6/JFH-1) were centrifuged at 1,000× rpm for 10 minutes to remove cells followed by another spin at 2,000× rpm for 15 minutes to remove cellular debris. Exosomes were positively selected with CD63 immunomagnetic beads as described above and the flow through collected which included cell free virus and viral particles. Levels of APOE and APOB proteins in the exosomes were identified by using Apolipoprotein E (APOE) Human ELISA Kit (Abcam cat # ab108813) and Human Apolipoprotein B (APOB) Quantikine ELISA Kit (R&D Systems cat # DAPB00) according to the manufacturers' protocols. The same number of control exosomes (obtained from non-infected Huh 7.5 cells), exosomes derived from HCV infected Huh 7.5 cells and viral particles were used for the experiment and quantified by Nanosight measurements. The optical density of the color reactions for both plates was read on plate reader at 450 nm. Standard curves were generated and concentrations of APOE and APOB were calculated as stipulated in the manufacturer's protocol. Liver cell protein lysate was used as positive control.

### Statistical analysis

Data are representative of at least 3 independently repeated experiments presented as mean + standard error of the mean (SEM). A non-parametric Mann-Whitney U test and multiple comparisons for repeated-measures were done using ANOVA performed with GraphPad Prism Version 5.0 (GraphPad Software). A p value of <0.05 was considered significant.

## Supporting Information

Figure S1 **Size distribution of CD63 immuno-isolated exosomes.** (A) Electron micrograph of CD63 positively selected exosomes from culture supernatants of Huh7.5 cells representing a size range from 50 nm to 100 nm. (B) Histogram plot of exosomes isolated from Huh7.5 cell culture supernatants were analyzed using NanoSight. (C) Histogram plot of exosomes isolated from sera of HCV infected patients were analyzed using NanoSight. Both exosomes derived from Huh7.5 cells and patients' sera were in the range of 50–100 nm. Data presented here is representative of 3 independent experiments.
(TIF)

Figure S2 **Free HCV virus and HCV exosomes are resistant to RNase treatment.** Free HCV virus, HCV CD63-immuno-selected exosomes and culture supernatant of HCV J6/JFH-1infected Huh7.5 cells were treated with RNase H (1 IU/ml &5 IU/ml) for 30 min then co-cultured with the naïve Huh7.5 cells as indicated. Total RNA was then extracted from cells 48 h after infection and assessed for HCV RNA by real-time quantitative PCR. Results are representative of 3 independent experiments.
(TIF)

Figure S3 **Type 1 interferon does not modulate exosome release from hepatocytes.** Huh7.5 cells were treated with different concentrations of interferon alpha as indicated over 48 h. Culture supernatants were then recovered and total exosomes isolated as decribed in the methods and quantified using NanoSight. Results are representative of 3 independent repeat experiments.
(TIF)

Figure S4 **Exosomes from HCV infected Huh 7.5 cells can transmit HCV to the naive Huh 7.5 cells.** Cell free supernatants from HCV-exosome infected Huh7.5 cells for conditions indicated above were used to infect Huh7.5 cells for 24 h alongside appropriate controls. Cells were then analyzed by western blot for HCV NS3 protein. Results are representative of 3 independent experiments.
(TIF)

Figure S5 **Specificity of HCV positive and negative sense RNA detection.** (A&B) Total RNA was extracted from free HCV virus and HCV J6/JFH-1 infected Huh7.5 cells. Total RNA was then reverse transcribed to cDNA using BioRad iScript cDNA Synthesis Kit. Using specific PCR conditions, as detailed in the methods, end point PCR products were run on a 1% agarose gel with ethidium bromide. Amplified PCR products were visualised using the BioRad ChemiDoc XRS Gel Photo Documentation System.
(TIF)

Figure S6 **Lansoprazole and bafilomycin A1 LDH toxicity assay.** (A&B) Lansoprazole and bafilomycin A1 toxicity was assessed in Huh7.5 cells after 24 h exposure at concentrations administered to the cells, using the LDH assay kit from Abcam according to the manufacturers specification. There was no statistically significant difference between cytotoxicity induced by different concentrations of bafilomycin A1(12.5 nM, 25 nM, and 50 nM) and untreated cells (p<0.001). There was no statistically significant difference between cytotoxixity induced by different concentration of Lansoprazole (5 µg/ml, 10 µg/ml and 50 µg/ml) and untreated cells (p<0.001). Staurosporine (20 nM) was used as a positive control and induced significant cell death. Results are representative of 4 repeat experiments with p<0.05 considered statistically significant by Mann Whitney U test.
(TIF)
