# Supplementary figures and images for: Exosomes from Hepatitis C Infected Patients Transmit HCV Infection and Contain Replication Competent Viral RNA in Complex with Ago2-miR122-HSP90
Source: PLoS Pathog. 2014 Oct 2;10(10):e1004424. doi: 10.1371/journal.ppat.1004424 (PMC4183590; doi:10.1371/journal.ppat.1004424)

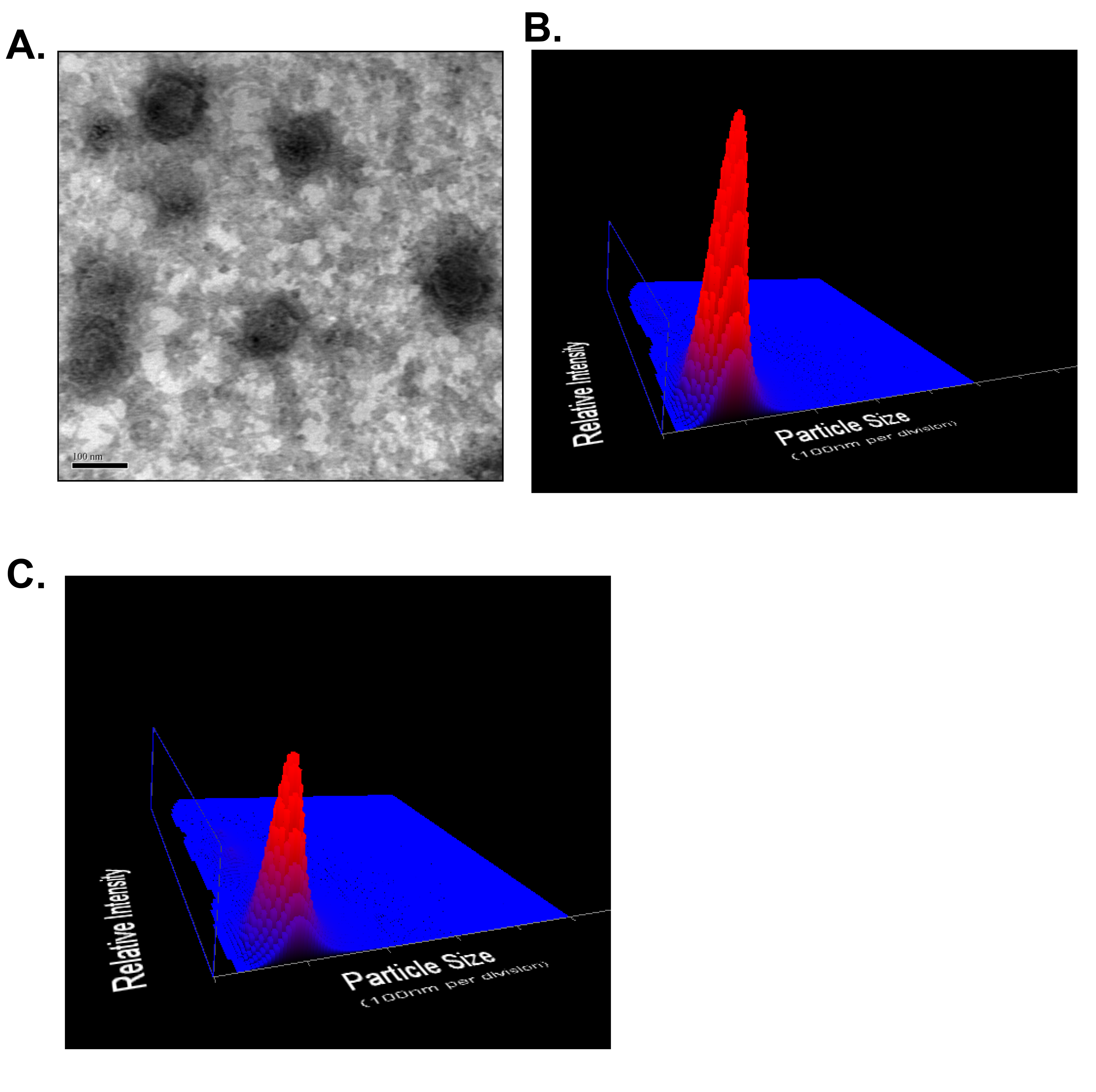

Supplement: Figure S1 — Size distribution of CD63 immuno-isolated exosomes. (A) Electron micrograph of CD63 positively selected exosomes from culture supernatants of Huh7.5 cells representing a size range from 50 nm to 100 nm. (B) Histogram plot of exosomes isolated from Huh7.5 cell culture supernatants were analyzed using NanoSight. (C) Histogram plot of exosomes isolated from sera of HCV infected patients were analyzed using NanoSight. Both exosomes derived from Huh7.5 cells and patients' sera were in the range of 50–100 nm. Data presented here is representative of 3 independent experiments. (TIF) [file ppat.1004424.s001.tif]

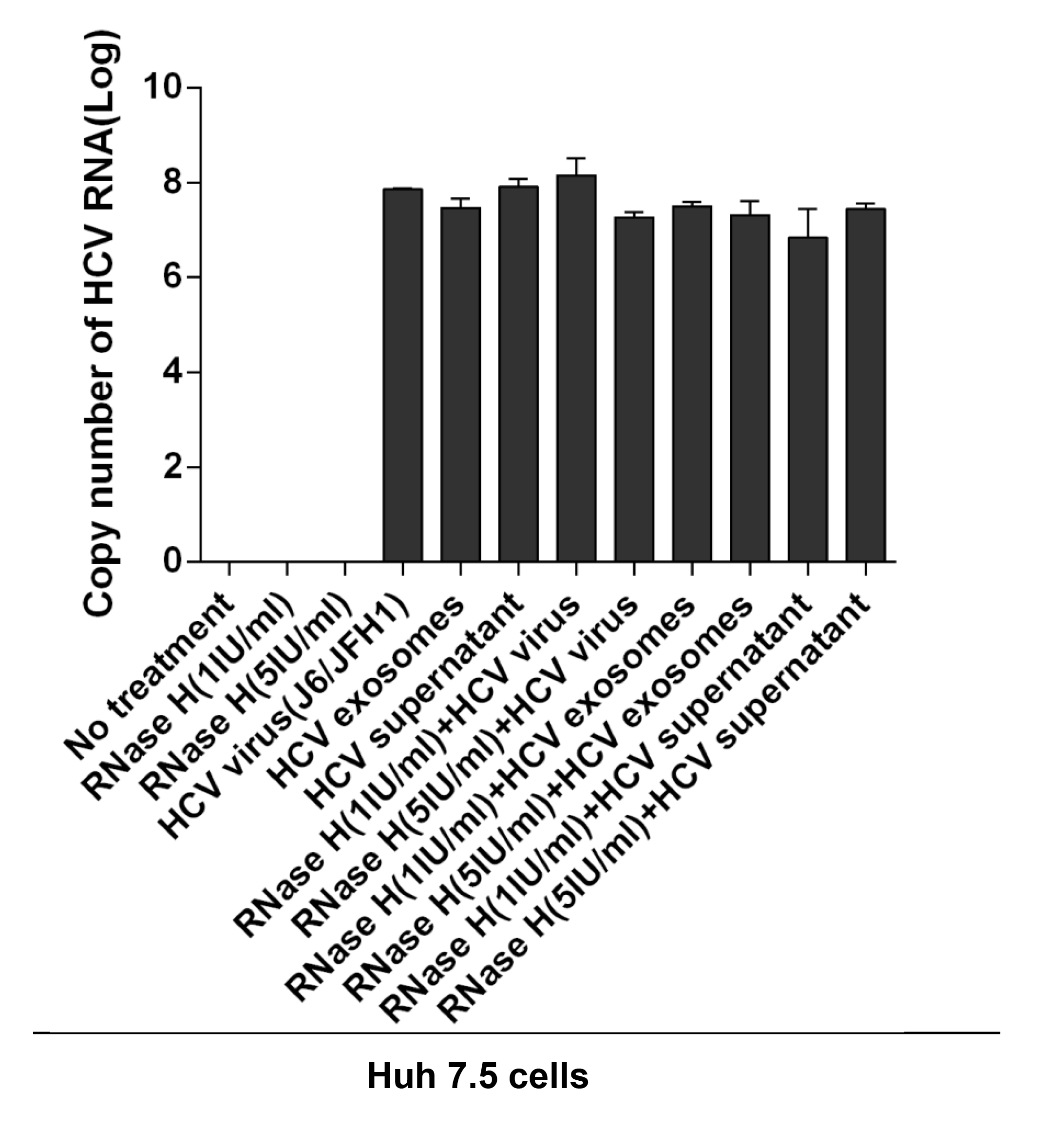

Supplement: Figure S2 — Free HCV virus and HCV exosomes are resistant to RNase treatment. Free HCV virus, HCV CD63-immuno-selected exosomes and culture supernatant of HCV J6/JFH-1infected Huh7.5 cells were treated with RNase H (1 IU/ml &5 IU/ml) for 30 min then co-cultured with the naïve Huh7.5 cells as indicated. Total RNA was then extracted from cells 48 h after infection and assessed for HCV RNA by real-time quantitative PCR. Results are representative of 3 independent experiments. (TIF) [file ppat.1004424.s002.tif]

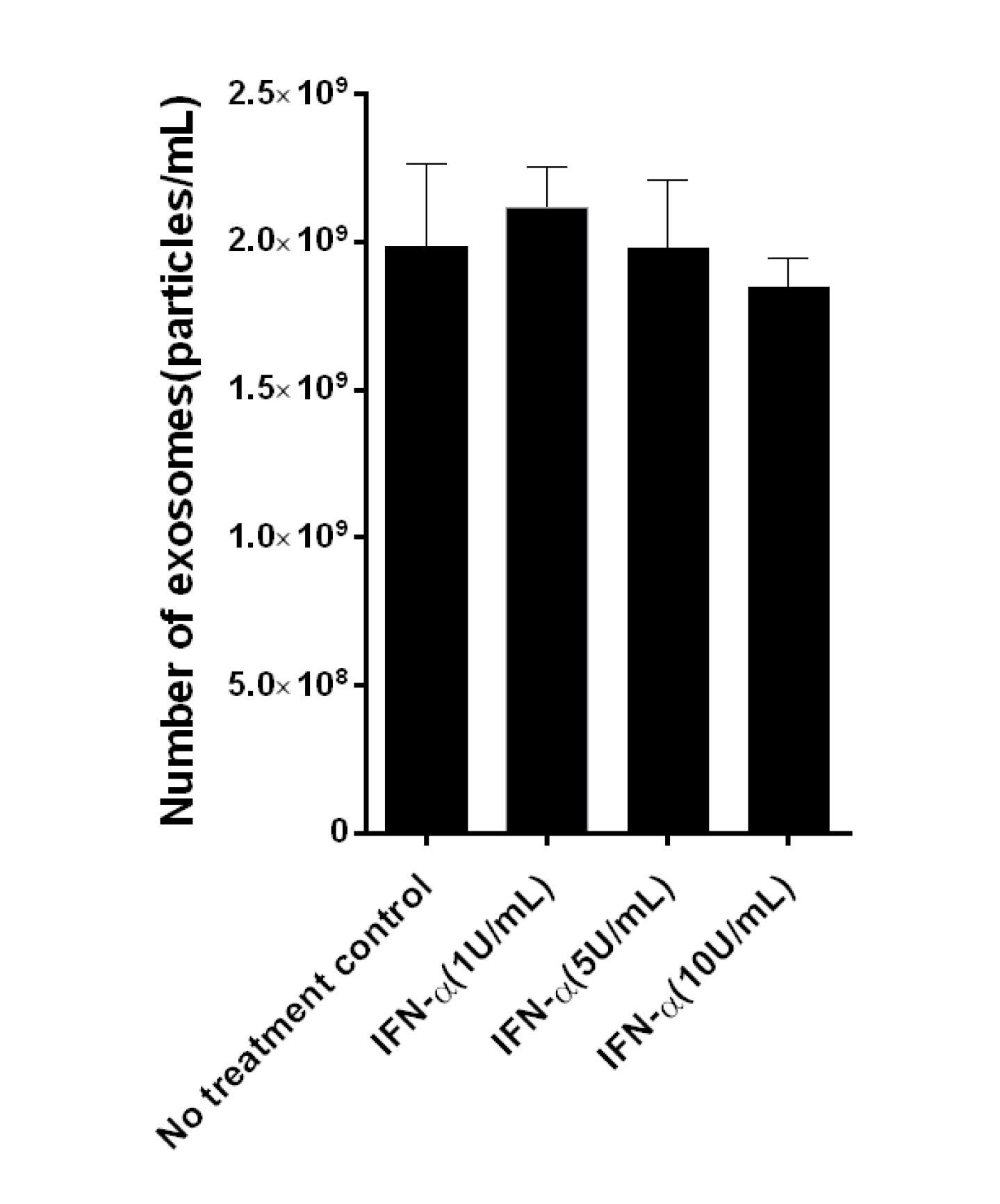

Supplement: Figure S3 — Type 1 interferon does not modulate exosome release from hepatocytes. Huh7.5 cells were treated with different concentrations of interferon alpha as indicated over 48 h. Culture supernatants were then recovered and total exosomes isolated as decribed in the methods and quantified using NanoSight. Results are representative of 3 independent repeat experiments. (TIF) [file ppat.1004424.s003.tif]

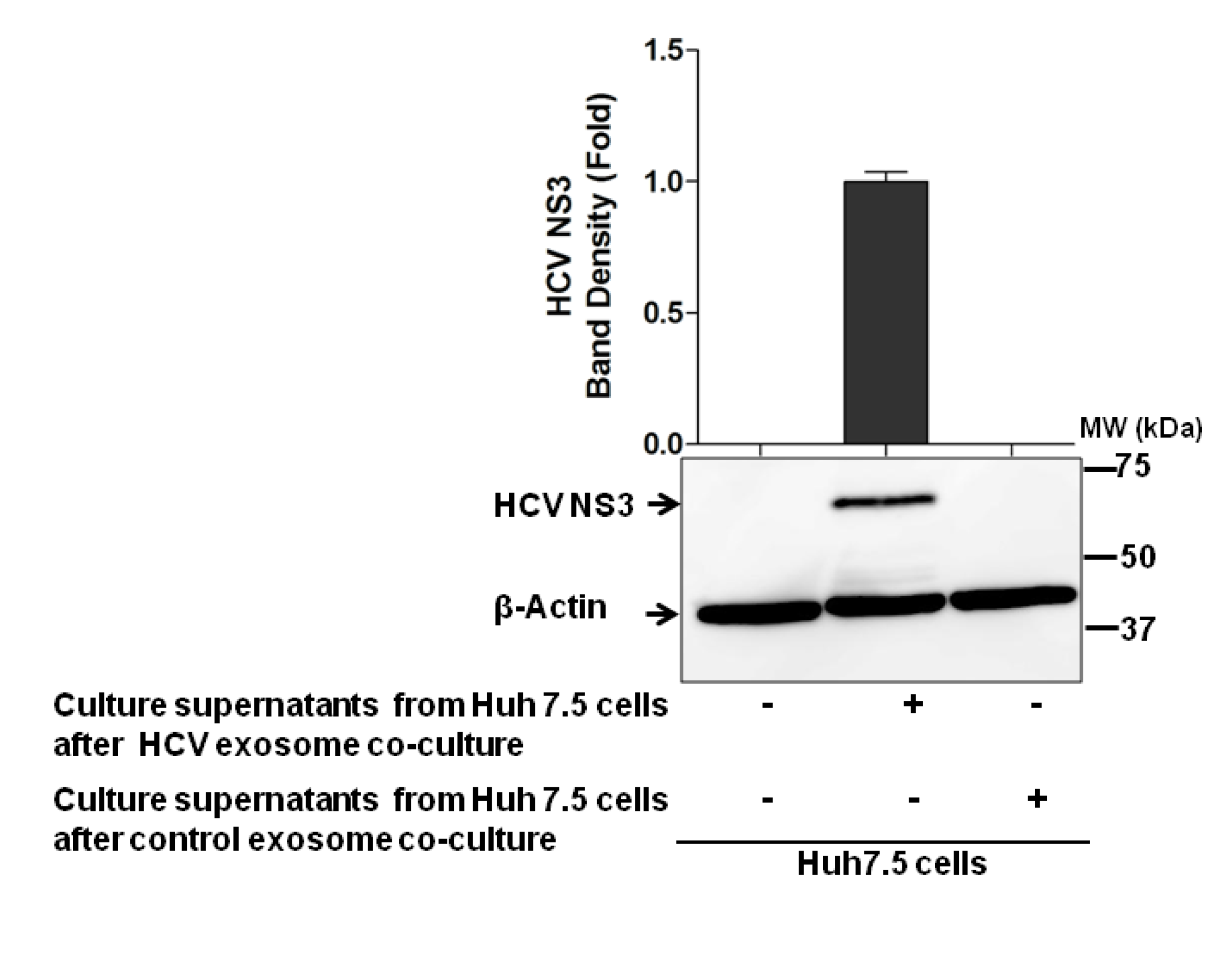

Supplement: Figure S4 — Exosomes from HCV infected Huh 7.5 cells can transmit HCV to the naive Huh 7.5 cells. Cell free supernatants from HCV-exosome infected Huh7.5 cells for conditions indicated above were used to infect Huh7.5 cells for 24 h alongside appropriate controls. Cells were then analyzed by western blot for HCV NS3 protein. Results are representative of 3 independent experiments. (TIF) [file ppat.1004424.s004.tif]

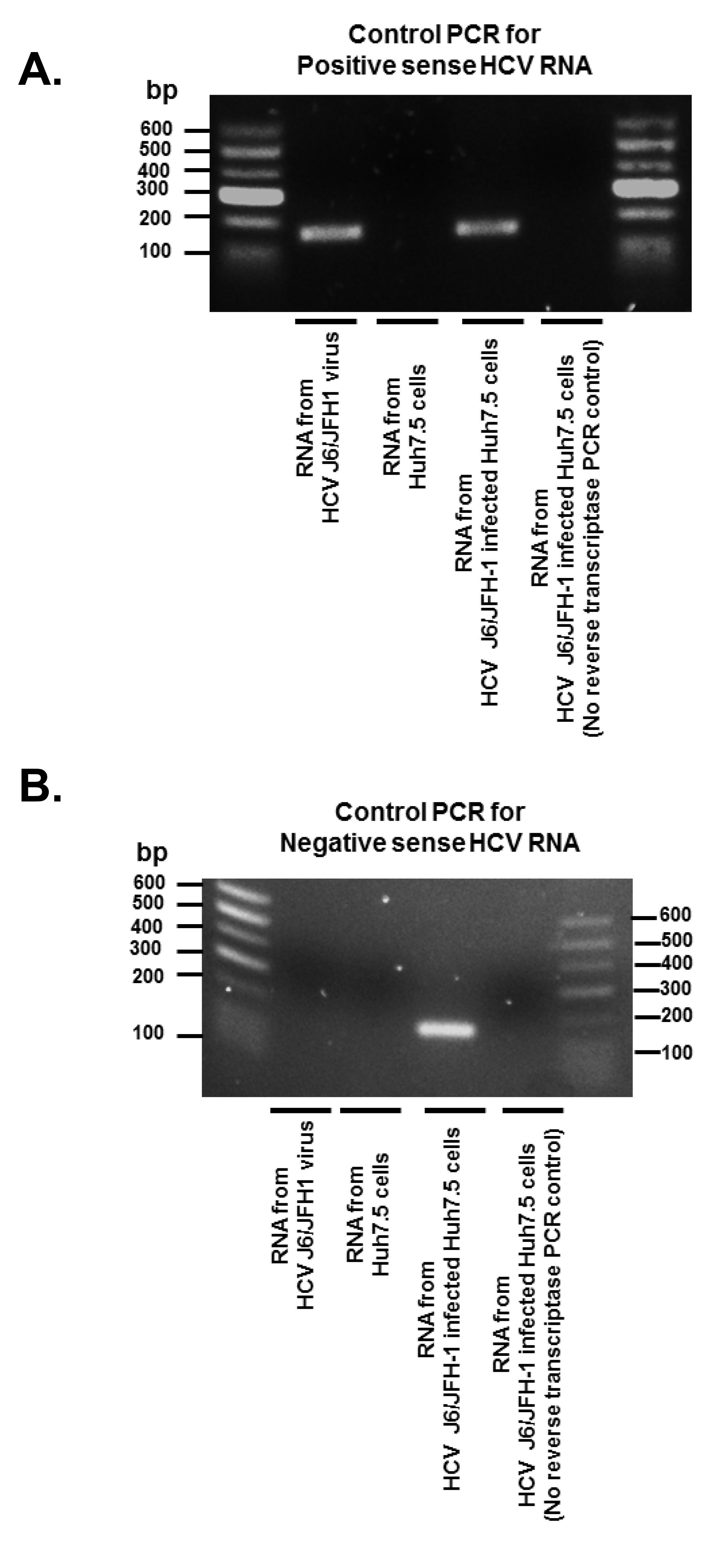

Supplement: Figure S5 — Specificity of HCV positive and negative sense RNA detection. (A&B) Total RNA was extracted from free HCV virus and HCV J6/JFH-1 infected Huh7.5 cells. Total RNA was then reverse transcribed to cDNA using BioRad iScript cDNA Synthesis Kit. Using specific PCR conditions, as detailed in the methods, end point PCR products were run on a 1% agarose gel with ethidium bromide. Amplified PCR products were visualised using the BioRad ChemiDoc XRS Gel Photo Documentation System. (TIF) [file ppat.1004424.s005.tif]

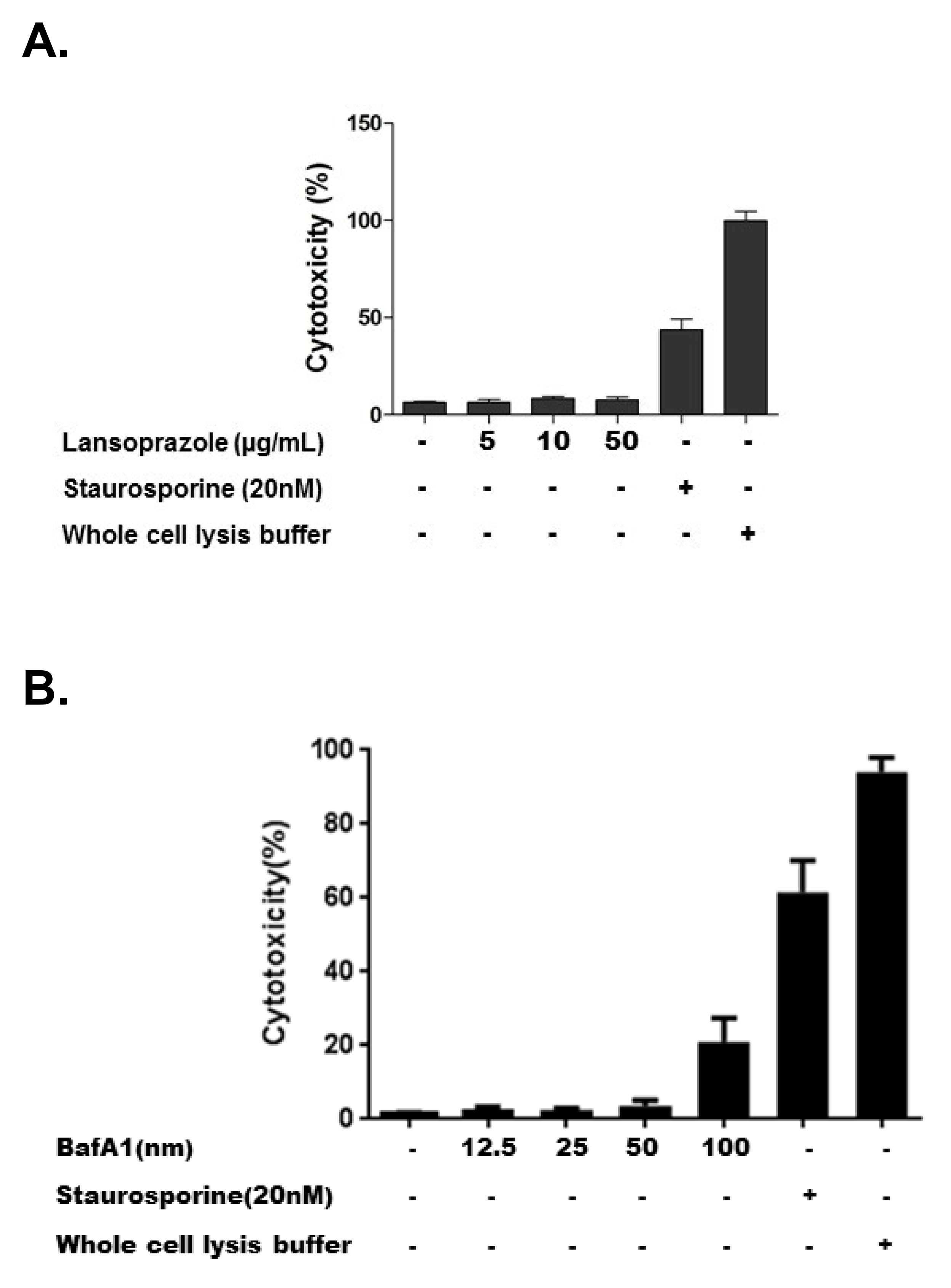

Supplement: Figure S6 — Lansoprazole and bafilomycin A1 LDH toxicity assay. (A&B) Lansoprazole and bafilomycin A1 toxicity was assessed in Huh7.5 cells after 24 h exposure at concentrations administered to the cells, using the LDH assay kit from Abcam according to the manufacturers specification. There was no statistically significant difference between cytotoxicity induced by different concentrations of bafilomycin A1(12.5 nM, 25 nM, and 50 nM) and untreated cells (p<0.001). There was no statistically significant difference between cytotoxixity induced by different concentration of Lansoprazole (5 µg/ml, 10 µg/ml and 50 µg/ml) and untreated cells (p<0.001). Staurosporine (20 nM) was used as a positive control and induced significant cell death. Results are representative of 4 repeat experiments with p<0.05 considered statistically significant by Mann Whitney U test. (TIF) [file ppat.1004424.s006.tif]
